# A systematic review and meta-analysis of CD22 CAR T-cells alone or in combination with CD19 CAR T-cells

**DOI:** 10.3389/fimmu.2023.1178403

**Published:** 2023-04-27

**Authors:** Nathan J. Fergusson, Komal Adeel, Natasha Kekre, Harold Atkins, Kevin A. Hay

**Affiliations:** ^1^ Department of Medicine, University of Toronto, Toronto, ON, Canada; ^2^ Clinical Epidemiology Program, Ottawa Hospital Research Institute, Ottawa, ON, Canada; ^3^ Faculty of Medicine, University of British Columbia, Vancouver, BC, Canada; ^4^ School of Epidemiology and Public Health, University of Ottawa, Ottawa, ON, Canada; ^5^ Division of Hematology, Department of Medicine, The Ottawa Hospital, Ottawa, ON, Canada; ^6^ Terry Fox Laboratory, BC Cancer Research Institute, Vancouver, BC, Canada; ^7^ Vancouver General Hospital, Leukemia and Bone Marrow Transplant Program of British Columbia, Vancouver, BC, Canada

**Keywords:** CAR T-cell, CD22, B-cell malignancies, efficacy, safety, systematic review & meta-analysis

## Abstract

Chimeric antigen receptor (CAR) T-cells are an emerging therapy for the treatment of relapsed/refractory B-cell malignancies. While CD19 CAR-T cells have been FDA-approved, CAR T-cells targeting CD22, as well as dual-targeting CD19/CD22 CAR T-cells, are currently being evaluated in clinical trials. This systematic review and meta-analysis aimed to evaluate the efficacy and safety of CD22-targeting CAR T-cell therapies. We searched MEDLINE, EMBASE, Web of Science, and the Cochrane Central Register of Controlled Trials from inception to March 3rd 2022 for full-length articles and conference abstracts of clinical trials employing CD22-targeting CAR T-cells in acute lymphocytic leukemia (ALL) and non-Hodgkin’s lymphoma (NHL). The primary outcome was best complete response (bCR). A DerSimonian and Laird random-effects model with arcsine transformation was used to pool outcome proportions. From 1068 references screened, 100 were included, representing 30 early phase studies with 637 patients, investigating CD22 or CD19/CD22 CAR T-cells. CD22 CAR T-cells had a bCR of 68% [95% CI, 53-81%] in ALL (n= 116), and 64% [95% CI, 46-81%] in NHL (n= 28) with 74% and 96% of patients having received anti-CD19 CAR T-cells previously in ALL and NHL studies respectively. CD19/CD22 CAR T-cells had a bCR rate of 90% [95% CI, 84-95%] in ALL (n= 297) and 47% [95% CI, 34-61%] in NHL (n= 137). The estimated incidence of total and severe (grade ≥3) CRS were 87% [95% CI, 80-92%] and 6% [95% CI, 3-9%] respectively. ICANS and severe ICANS had an estimated incidence of 16% [95% CI, 9-25%] and 3% [95% CI, 1-5%] respectively. Early phase trials of CD22 and CD19/CD22 CAR T-cells show high remission rates in ALL and NHL. Severe CRS or ICANS were (1)rare and dual-targeting did not increase toxicity. Variability in CAR construct, dose, and patient factors amongst studies limits comparisons, with long-term outcomes yet to be reported.

**Systematic review registration:**

https://www.crd.york.ac.uk/prospero, identifier CRD42020193027.

## Introduction

The treatment of relapsed/refractory (R/R) B-cell malignancies remains a challenge. Among patients with B-cell acute lymphoblastic leukemia (B-ALL) who have failed standard induction chemotherapy, only 45% achieve complete remission with salvage chemotherapy, and one-year survival is only 26% (1). Likewise, in diffuse large B-cell lymphoma (DLBCL), patients with refractory disease have a CR rate of 7% with salvage chemotherapy and a one-year survival of 28% (2). The emergence of chimeric antigen receptor (CAR) T-cell immunotherapy, in which T-cells are genetically engineered to express CARs targeting specific tumor-associated antigens, has significantly changed the treatment of these R/R diseases. CD19 CAR T-cells, the most well-established B-cell antigen target, demonstrated promising responses in clinical trials, with CR rates of 70-90% in B-ALL and 50% in certain Non-Hodgkin’s lymphoma (NHL) patients (3–6). Today, there are four FDA-approved CD19 CAR T-cell therapies approved for use in NHL (axi-cel, tisa-cel, liso-cel, and brexu-cel), with brexu-cel now also approved for use in patients under 25 with ALL (6–14).

Despite these impressive results, around 30% of patients fail to respond to CD19 CAR T-cells, and 36-57% of patients who achieve CR relapse within one year (5, 11, 15). In particular, a subset of relapses are associated with loss of CD19 expression or escape splice variants on malignant cells, with prior studies finding 16-68% of relapses to be CD19-negative (11, 15, 16). Alternate CAR targets for B-cell malignancies are now being explored, including CD22, CD20, CD79b and BAFF-R (17–23). Among these, CD22 has been the focus of a large number of clinical trials in recent years, both as a single target and as part of dual-targeting CD19/CD22 CAR T-cells. One theoretical advantage of dual-targeting is the prevention of antigen-negative relapse.

The recent influx of CD22 and CD19/CD22 CAR T-cell therapies entering clinical trials warrants a systematic review to evaluate their efficacy and to assess the risk of adverse events that are common in CAR T-cell therapies, such as cytokine release syndrome (CRS) and immune effector cell associated neurotoxicity syndrome (ICANS). There is limited synthesis of clinical trial findings, with the majority of prior systematic reviews focusing on CD19 CAR T-cells (24–26). Grigor et al. and Yu et al. conducted broader reviews of all CAR T-cells, but only included studies up to late 2017 and early 2018, respectively, and as such both only include one CD22 CAR T-cell trial (5, 27). Li et al. is the only systematic review we identified that focused on CD22 and/or CD19/CD22 CAR T-cell therapies, however, it only included ten studies (28). We conducted a preliminary scan and found that a significant amount of CD22 CAR T-cell clinical trial data is currently published only in the form of conference abstracts, which were not included in the review by Li et al. As such, a meta-analysis including data from abstracts will provide a more comprehensive review of current findings.

We conducted a systematic review and meta-analysis of CAR T-cells targeting CD22, alone or in combination with other antigen targets, to evaluate their efficacy and safety in the treatment of patients with B-cell malignancies.

## Methods

### Registration

This systematic review and meta-analysis was conducted in accordance with the PRISMA guidelines (details in **Supplementary Materials**) (29). The protocol was prospectively registered in PROSPERO (CRD42020193027), and the full protocol is published in a peer-reviewed journal (30).

### Eligibility criteria, data sources and search strategy

Interventional studies, with or without a comparator, on CD22 CAR T-cell therapy in patients with B-cell malignancies were eligible for inclusion. This included studies investigating multi-target therapies, such as multi-targeted CD19 and CD22 CAR T-cells (CD19/CD22 CAR T-cell therapy). Only studies that reported the primary outcome of interest, complete response (CR), were included. Full-length articles, conference abstracts, letters and case reports were considered, while reviews, editorials, and commentaries were excluded. Studies for which an associated clinical trial could not be identified (using a clinical trial registration number) were excluded to avoid double-counting participants.

MEDLINE, EMBASE, Web of Science, and the Cochrane Central Register of Controlled Trials were searched from inception to March 3 2022. Additionally, the conference proceedings of the American Society of Hematology, American Society of Clinical Oncology, and European Hematology Association were searched manually. Bibliographies of all included studies were also searched. The search strategy was created in collaboration with an experienced health science librarian. No language restrictions were applied. The full search strategy can be found in the **Supplementary Materials**. In addition to the search of study reports, ClinicalTrials.gov and the WHO International Clinical Trials Registry Platform (ICTRP) were searched to catalogue any relevant registered clinical trials.

### Study selection

Search results were uploaded to Covidence systematic review software (Veritas Health Innovation, Melbourne, Australia). Title and abstract screening, full-text screening, data extraction and risk of bias assessment were conducted by two reviewers in duplicate (N.J.F and K.A). Disagreements were resolved by discussion or a third reviewer (K.A.H. and H.A.). Within included reports, multiple reports of the same study were identified and grouped by associated clinical trial number. For each study, the most recent full-length article was used as the primary report for data extraction, with any other reports cross-referenced for **Supplemental Information**. If no full-length article existed for a given study, the most recent conference abstract or case report was used as the primary report.

### Data items and extraction

A piloted form on Covidence was used for data extraction. Publication, study, patient and intervention characteristics as well as manufacturing, efficacy, and safety outcomes were extracted. Health Related Quality of Life (HRQoL) or Patient-Reported Outcomes (PRO) were also sought.

The primary outcome extracted for meta-analysis was best CR rate (bCR), defined as the proportion of patients reported to have achieved CR at any point during follow-up; one-month CR and three-month CR rate were also extracted. Secondary efficacy outcomes included overall response, relapse rate, and time-to-event data (overall survival and progression-free survival). Safety outcomes included reported incidence of adverse events (CRS, ICANS, graft-versus-host disease, infection, and other reported adverse events) and 30-day mortality rate. Full details of these and other data items are available in the previously published protocol (30). Study quality was assessed using a modified Institute of Health Economics (IHE) risk of bias tool (31).

### Data synthesis and analysis

Meta-analysis was deemed appropriate for bCR, CRS, and ICANS; other outcomes are synthesized narratively as there was significant variation in reporting by included studies. Meta-analyses were conducted using R statistical software (v.4.2.2). Binary outcomes are presented as proportions with 95% confidence intervals (CI). A random effects model (DerSimonia and Laird) was employed to pool proportions using an arcsine-based transformation (metaprop function from R package metafor). Given the prevalence of low and high event rates in our data, an arcsine transformation was deemed more appropriate than a logit transformation (32). Cochrane I^2^ statistic is used to assess statistical heterogeneity between summary data.

Subgroup analysis was undertaken using a meta-regression technique (metareg function from the R package metafor). Pre-specified subgroups of interest were malignancy type, single vs. dual targeting therapy, age group, and previous therapy (previous transplant, previous CD19 CAR T-cell therapy, previous non-CAR-T cell immunotherapy). A sensitivity analysis was performed by removing data from conference abstracts and evaluating the effect on the results. An alternative funnel plot (study size vs arcsine transformed outcome proportion) was used to assess publication bias (33). The GRADE approach was used to evaluate confidence in treatment effects (34).

## Results

### Results of search

**Figure 1:**
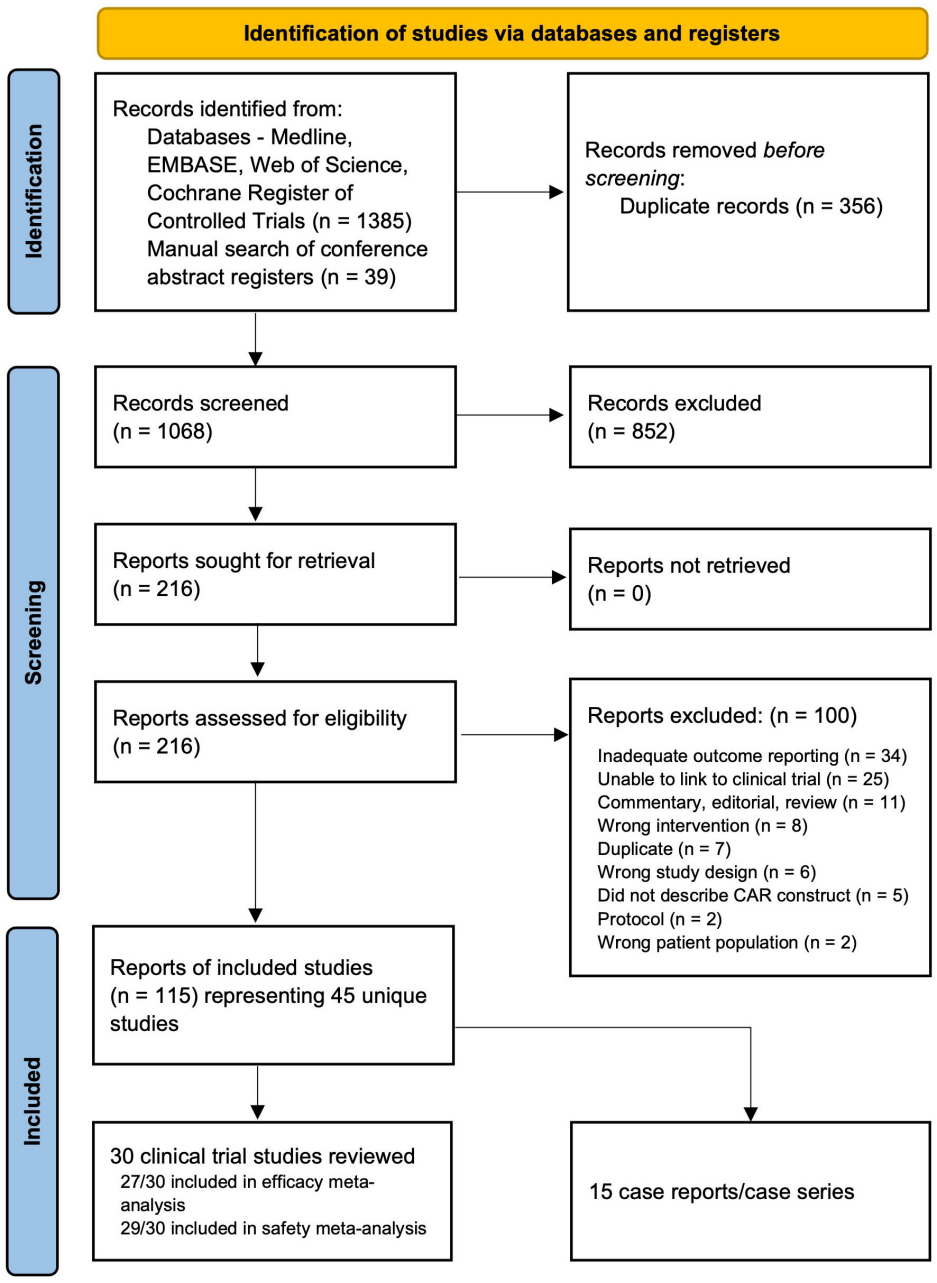
PRISMA flow diagram of references identified, screened, excluded (with reasons) and included.

From 1068 unique references, 115 references were included in our review, representing 45 unique studies (**Figure 1**) (35–79). Among these, 29 were eligible for meta-analysis (27 studies with a total of 578 patients for efficacy analysis and 29 studies with a total of 637 patients for safety analysis); and 15 were treated as case reports/series. Of note Liu 2021A was excluded from both the response and safety meta-analyses due to heterogeneity in study design and insufficient safety data, however is summarized narratively and in our tabular data synthesis. Details of the studies included in the meta-analysis are presented in **Table 1**. All studies were early-phase single-arm clinical trials. A full list of included references grouped by study can be found in **Supplementary Materials**. Outcomes from case reports and case series are summarized separately in **Supplementary Table 1**.

**Table 1:** Study Characteristics of Included Clinical Trials.

Study ID	Clinical Trial No.	Country	Phase	Publication Type	Malignancy	Antigen Target
Annesley 2021	NCT03330691	USA	1	Abstract	ALL	CD19/CD22
Baird 2021	NCT04088890	USA	1	Letter	LBCL	CD22
Cao 2021	ChiCTR-OPN-16009847	China	0	Full Report	NHL	CD19/CD22
Cordoba 2021	NCT03289455	UK	1	Full Report	ALL	CD19/CD22
Dai 2020	NCT03185494	China	1	Full Report	ALL	CD19/CD22
Frey 2021	NCT03620058	USA	1	Abstract	ALL	CD19/CD22
Gardner 2020	NCT03330691	USA, Canada	1	Abstract	ALL	CD19/CD22
Hu 2021	NCT04227015	China	1	Full Report	ALL	CD19/CD22
Liu 2021 A	ChiCTR-ONC-17013648	China	1	Full Report	ALL	CD19/CD22
Liu 2021 B	NCT03614858	China	1/2	Abstract	ALL	CD19/CD22
Liu 2022	ChiCTR1800014457	China	1	Full Report	NHL	CD19/CD22/ CD20
Pan 2019	ChiCTR-OIC-17013523	China	1	Full Report	ALL	CD22
Pan 2020	ChiCTR-OIB-17013670	China	1	Letter	ALL	CD19/CD22
Ramakrishnan 2020	NCT03287817	UK, USA	1	Abstract	DLBCL	CD19/CD22
Schultz 2018	NCT03241940	USA	1	Abstract	ALL	CD19/CD22
Shah 2020	NCT02315612	USA	1	Full Report	ALL	CD22
Shalabi 2020	NCT03448393	USA	1	Abstract	ALL	CD19/CD22
Singh 2021^†^	NCT02650414 and NCT02588456	USA	1	Full Report	ALL	CD22
Speigel 2021	NCT03233854	USA	1	Full Report	ALL, LBCL	CD19/CD22
Summers 2021^††^	NCT03244306 (V1) and NCT04571138 (V2)	USA	1	Abstract	ALL	CD22
Tan 2021	ChiCTR2000028793	China	1	Letter	ALL	CD22
Wang 2020	ChiCTR-OPN-16008526	China	1	Full Report	ALL, NHL	CD19/CD22
Wang 2021	ChiCTR2000032211	China	0	Full Report	ALL	CD19/CD22
Wei 2021	ChiCTR1800015575	China	1	Full Report	ALL, NHL	CD19/CD22
Yang 2018	NCT03312205	China	1	Abstract	ALL	CD19/CD22
Yang 2020	NCT04129099	China	1	Abstract	ALL	CD19/CD22
Yang 2019	NCT03825731	China	1	Abstract	ALL	CD19/CD22
Zhang 2021 A	NCT03196830	China	2	Full Report	NHL	CD19/CD22
Zhang 2021 B	NCT04539444	China	2	Abstract	NHL	CD19/CD22
Zhu 2021	ChiCTR1800019298	China	1	Full Report	ALL, DLBCL	CD22

ALL, acute lymphoblastic leukemia; NHL, Non-Hodgkin’s Lymphoma; LBCL, large B-cell lymphoma; DLBCL, diffuse large B-cell lymphoma. ^†^Singh 2021 reports combined results of an adult and pediatric study investigating the same intervention. ^††^Summers 2021 reports results of an initial (V1) and optimized (V2) CAR construct.

### Patient characteristics

The majority of studies examined R/R B-ALL patients as the population of interest, however a significant subset of studies examined R/R NHL. Among the seven CD22 CAR T-cell studies, five included only B-ALL patients, one included B-NHL, and one examined both B-ALL and B-NHL. Among the 23 studies examining CD19/CD22 CAR T-cells, 15 included only B-ALL patients, 5 included B-NHL patients, and 3 included both B-ALL and B-NHL patients. Patient demographics, disease status, and prior therapies are presented in **Table 2**. Among CD22 CAR T-cell studies that provided information on prior CAR T-cell therapy (5 out of 7 studies), the majority of patients had received prior CD19 CAR T-cells (108/137, 79%). This includes Zhu 2021 who included only patients who had relapsed post-CD19 CAR T-cells. In comparison, prior CD19 CAR T-cell therapy was less commonly reported in CD19/CD22 CAR T-cell studies, with only 11% of patients (20/176) having had prior CD19 CAR-T cell therapy among 11 studies, with the remaining 12 studies not providing information on prior CAR T-cell therapies. Many patients had received other prior therapies, including hematopoietic stem cell transplant (HCT) and targeted immunotherapies such as blinatumomab and inotuzumab.

**Table 2:** Intervention Characteristics of Included Clinical Trials.

Study ID	Method of co-targeting	sCFV domain	Costimulatory Domain	T-cell source	CAR T-cell Dose in cells/kg (except where marked * indicating non-weight based dose)
CD22					
Pan 2019	N/A	YK-CD22	41BB	Auto/allo^†^	Median 5 x10^5^, range 0.2-34.7
Shah 2020	N/A	m971	41BB	Auto	DL1: 3x10^5^, DL2: 1x10^6^, DL3: 3x10^6^
Singh 2021	N/A	m971 (long linker)	41BB	Auto	Patients <50 kg: 1–10 x 10^6^ cells/kg. Patients >50 kg: 5.0 x 10^8^ cells total* in fractioned adaptive dosing scheme.
Summers 2021	N/A	m971	41BB	Auto	V1 DL1: 1 x 10^6^, DL2: 3 x 10^6^ V2: 2 x 10^5^
Tan 2021	N/A	FH80	41BB	Auto	Median: 1.2 x 10^6^, range 0.68-9.4
Zhu 2021	N/A	NR	41BB	Auto	2.0 x 10^6^
Baird 2021	N/A	m971	41BB	Auto	DL1: 1 x 10^6^ (n = 12), DL2: 3 x 10^6^ (n = 9)
CD19/CD22					
Annesley 2021	Co-transduction	NR	CD19: 41BB CD22: 41BB	Auto	DL1: 0.5 x 10^6^ (n = 3) DL2: 1 x 10^6^ (n = 3) DL3: 3 x 10^6^ (n =6)
Cordoba 2021	Bicistronic vector	Humanized	CD19: OX40 CD22: 41BB	Auto	DL1: 1x10^6^ (n = 2) DL2: 3x10^6^ (n = 5) DL3: 5x10^6^ (n = 5)
Dai 2020	Bivalent CAR	CD19: FMC63 CD22: m971	41BB	Auto	Mean: 2.28 x10^6^ (range: 1.7-3)
Frey 2021	Co-administration	Humanized	CD19: 41BB CD22: 41BB	Auto	Planned CD19 dose: 2.0 x 10^6^ Planned CD22 dose: 2.0 x 10^6^ With fractionated adaptive dosing scheme.
Gardner 2020	Co-transduction	CD19: FMC63 CD22: m971	CD19: 41BB CD22: 41BB	Auto	DL1: 1x10^6^ DL2: 3x10^6^
Hu 2021	Bivalent CAR	CD19: FMC63 CD22: m971	41BB	Allo (UCART)^††^	DL1: 1 x 10^6^ DL2: 3 x 10^6^
Liu 2021 A	Sequential infusion (months)	CD19: FMC63 CD22: Human phage library	CD19: 41BB CD22: 41BB	Auto	Median CD19: 1.0 x 10^5^ (range 0.486 - 5.0) Median CD22: 2.0 x 10^5^ (range 0.32 - 5.0)
Liu 2021 B	Group 1: Tandem Group 2: Sequential	NR	NR	NR	NR
Pan 2020	Sequential infusion (months)	CD19: FMC63 CD22: YK-CD22	CD19: 4-1BB CD22: 4-1BB	Auto	CD19: 10 x 10^5^ (range 3.3 - 42.8) CD22: 10 x 10^5^ (range, 0.25 - 47.4)
Schultz 2018	Bivalent CAR	CD19: FMC63 CD22: m971	41BB	Auto	DL1: 1 x 10^6^ Subsequent doses not yet reported
Shalabi 2020	Bivalent CAR	CD19: FMC63 CD22: m971	41BB	Auto	DL1: 3 x 10^5^ DL2: 1 x 10^6^ DL3: 3 x 10^6^
Speigel 2021	Bivalent CAR	CD19: FMC63 CD22: m971	41BB	Auto	DL1: 1 x 10^6^ DL2: 3 x 10^6^
Wang 2020	Sequential infusion (days)	NR	CD19: CD28, 4-1BB CD22: CD28, 4-1BB	Auto	ALL: CD19: 2.5 x 10^6^, CD22: 2.5 x 10^6^ NHL: CD19: 5 x 10^6^, CD22: 5x 10^6^
Wang 2021	Co-administration	NR	41BB	Auto	Mean CD19: 3.975 x 10^6^ (range 3-6) Mean CD22: 3.125 x 10^6^ (range 2-4)
Wei 2021	Bivalent CAR	CD19: FMC63 CD22: human phage library	41BB	Auto	BCL: Median 6.3 x 10^6^ (range 4.9-9.4) ALL: 4.85 x 10^6^ (range 1.04-7.02)
Yang 2018	Co-transduction	NR	CD19: CD28, 4-1BB CD22: CD28, 4-1BB	Auto	Median CD19: 2 x 10^5^, range 0.9-5 Median CD22: 0.5 x 10^5^, range 0.4-12
Yang 2020	Bivalent CAR	NR	41BB	Auto	DL1: 6.0 x 10^4^ (n = 2) DL2: 1.0-1.5 x 10^5^ (n=7) DL3: 2.25 x 10^5^ (n=1)
Yang 2019	Bivalent CAR	NR	41BB	Auto	DL1: 2.5-5 x 10^5^(n = 4) DL2: 1-2.5 x 10^6^ (n = 7) DL3: 3-5 x 10^6^ (n = 5)
Cao 2021	Sequential infusion (days) following HSCT	Murine	CD19: CD28, 41BB CD22: CD28, 41BB	Auto	Median CD19: 4.1 x 10^6^ (range 1.8 - 10) Median CD22: 4.0 x 10^6^ (range 1.0 - 10)
Liu 2022^§^	Sequential infusion (months) - CD22 only given after CD19 failure	CD22: Human phage library CD19: FMC63/human phage library	CD19: 41BB CD22: 41BB	Auto	Median CD19: 2.0 x 10^6^ (range 0.11 - 3.0) Median CD22: 2.0 x 10^6^ (range 0.17 - 4.13) Median CD20: 1.29 x 10^6^ (range 0.44 - 2.17)
Ramakrishnan 2020	Bicistronic vector	NR	CD19: OX40 CD22: 41BB	Auto	DL1: 50 x 10^6^ CAR T-cells total* DL2: 150 x 10^6^ CAR T-cells total* DL3: 450 x 10^6^ CAR T-cells total*
Zhang 2021 A	Bivalent CAR	CD19: FMC63 CD22: m971	41BB	Auto	Median: 8.258 x 10^8^ CAR T-cells total* (range 3.690 x 10^8 to 3.285 x 10^9^)
Zhang 2021 B*	Sequential infusion (days)	NR	CD19: 41BB CD22: 41BB	NR	0.5 - 2 x 10^7^

Co-transduction: Simultaneously transducing T-cells with two separate vectors. Bivalent CAR: A single CAR molecule that has two specificity domains. Bicistronic vector: transduction of a single vector that expresses two CARs. Sequential infusion: infusion of one CAR-T cell product followed by a different CAR-T cell product, either on successive days (days) or delayed (months). DL, dose level. Fractionated adaptive dosing scheme: dose is given in fractions over 3 days, and subsequent doses held if develop adverse events after first dose. ^†^Pan 2019: allogeneic cells were allowed in patients with previous transplant. ^††^Hu 2021 used CRISPR-Cas9 engineered universal CAR T-cells (donor-derived). ^§^Liu 2022 only gave CD22 after patients failed CD19, therefore not all patients received CD22. *In Zhang 2021, intervention included anti-PD-1 antibody administered after sequential infusion of CD19 and CD22 CAR-T cells. N/A, not applicable; NR, not reported.

### Intervention characteristics

There were seven studies of CAR T-cell therapy solely targeting CD22 (CD22 CAR T-cells), and 23 studies investigated CAR T-cell therapies targeting both CD19 and CD22 (CD19/CD22 CAR T-cells). This includes Liu 2022, in which select patients also received CD20 CAR T-cells if CD19 and CD22 CAR T-cells failed. Various dual-targeting methods were used, including bivalent CAR molecules, bicistronic vectors, co-administration, and sequential infusion of two CAR T-cell populations. Details are presented in **Table 3**. Cao 2021 was unique in that it involved sequential CD19/CD22 CAR T-cell infusion after all participants had received autologous HCT. Ramakrishnan 2020 and Zhang 2021B both combined CAR T-cells with anti-PD1 antibody therapy. Hu 2021 was unique because it utilized a universal donor-derived CAR T-cell product in which CRISPR/Cas9 was used to disrupt the TRAC region and CD52 gene of CAR T-cells to minimize host CAR T-cell rejection and to allow for anti-CD52-mediated targeted depletion of autologous T-cells.

**Table 3:** Characteristics of Treated Patients.

Study ID		Malignancy	N	Sex (% M)	Median age (range)	Prior CD19 CAR T-cells	Other prior therapy
CD22							
Pan 2019		ALL	34	59%	10 (1-55)	31/34	Allo-HCT: 13/34
Shah 2020		ALL	58	NR	17.5 (4.4-30.6)	36/58	CD22 CAR T cells: 5/58 Inotuzumab: 14/58 CD19-targeted therapy: 51/58 Blinatumomab: 23/58 HCT: 39/58
Singh 2021		ALL	8	NR	NR; 3 adults, 5 pediatrics	5/8	Prior allo-HSCT: 3/8 Blinatumomab: 3/8
Summers 2021	V1	ALL	4	NR	NR	NR	NR
	V2	ALL	3	NR	NR	3/3	NR
Tan 2021		ALL	8	25%	9 (5-16)	NR	Prior CD19 and CD22 directed therapies: 8/8 Prior allo-HSCT: 4/8
Zhu 2021		ALL	6	50%	39.5 (25-58)	13/13	NR
		DLBCL	7	71%	56 (16-70)		
Baird 2021		LBCL	21	62%	64 (36-79)	20/21	Allo-SCT: 6/21
CD19/CD22							
Annesley 2021		ALL	11	NR	NR	4/11	CD19 or CD22 targeted therapies: 11/12 enrolled pts
Cordoba 2021		ALL	15	73%	8 (4-16)	1/15	Allo-SCT: 7/15
Dai 2020		ALL	6	67%	23.5 (17-44)	NR	NR
Frey 2021		ALL	13	NR	46 (28-71)	2/13	Blinatumomab: 8/13 Inotuzumab: 8/13 Prior allogeneic SCT: 10/13
Gardner 2020		ALL	27	NR	NR	NR	CD19 or CD22 targeted therapies: 13/27
Hu 2021		ALL	6	33%	49 (26-56)	NR	NR
Liu 2021 A		ALL	27	52%	21 (1.6–55)	NR	All had relapsed post allo-HCT
Liu 2021 B	Tandem	ALL	49	NR	NR	NR	NR
	Sequential	ALL	13	NR	NR	NR	
Pan 2020		ALL	20	65%	6 (1-16)	NR	NR
Schultz 2018		ALL	4	NR	NR (2-17)	NR	NR
Shalabi 2020		ALL	11	NR	21 (5-28)	5/11	NR
Speigel 2021		ALL	17	71%	47 (26-68)	1/17	Allo-SCT: 12/17
		LBCL	21	67%	69 (25-78)	NR	Auto-SCT: 4/21
Wang 2020		ALL	51	63%	27 (9-62)	NR	Allo-HCT: 9/51 Auto-HCT: 3/51
		NHL	38	58%	47 (17-11)	NR	Auto-HCT: 6/38
Wang 2021		TCF3-HLF+ALL	4	100%	6.8 (2.9-14.7)	NR	NR
Wei 2021		ALL	15	47%	27 (16-65)	0/15	Prior HSCT: 1/15
		NHL	16	50%	52.5 (23-68)	0/16	Auto-HSCT: 1/16
Yang 2018		ALL	15	73%	19 (4-45)	NR	NR
Yang 2020		ALL	10	50%	11.5 (3-48)†	3/10	Allo-HSCT: 1/10
Yang 2019		ALL	16	59%	8 (4-45)	4/17	NR
Cao 2021		Aggressive NHL	42	57%	41 (24-61)	NR	No prior HSCT
Liu 2022		Burkitt	23	NR	8 (2-12)	NR	NR
Ramakrishnan 2020		DLBCL	19	NR	57 (28-71)	0/19	No prior CD19 or CD22-directed therapies or allo-HCT
Zhang 2021 A		NHL	32	59%	NR	0/32	Prior HSCT: 4/32
Zhang 2021 B		NHL	11	NR	NR	NR	NR

NR, not reported.

Thirteen of 30 studies were dose escalation trials, but only six identified a recommended expansion phase dose. Annesley 2021, Gardner 2020, and Spiegel 2020 all identified the recommended dose of CD19/CD22 CAR T-cells to be 3x10^6^ CAR T-cells/kg for ALL patients (and LBCL patients in Spiegel 2020), with no dose-limiting toxicities identified. Spiegel 2020 commented that they did not pursue higher doses due to toxicity concerns at higher doses seen in other clinical trials. Conversely, Cordoba 2021 treated ALL patients with up to 5x10^6^ CD19/CD22 CAR T-cells cells/kg. No dose-limiting toxicities were identified, and while CAR T-cell persistence was identified as an issue, higher doses were not pursued due to a lack of correlation between higher dose and persistence. Among CD22 CAR T-cell studies, Baird 2021 examined two dose levels of 1x10^6^ and 3x10^6^ CAR T-cells/kg for LCBL patients, however 1x10^6^ was the maximum tolerated dose. Shah 2020 also initially used 1x10^6^ CD22 CAR T-cells/kg in their expansion phase to treat ALL patients, but the dose was de-escalated to 3x10^5^ CAR T-cells/kg following increased toxicity with the institution of a CD4/CD8 selection procedure during manufacturing. In all studies except Ramakrishnan 2020, CAR T-cells were dosed by weight. A number of studies also used a fractionated adaptive dosing scheme, in which the target dose was given in split infusions, with subsequent infusions being held if toxicity developed.

### Response data

27 of 30 studies were included in meta-analysis of bCR rates. Cao 2018, Liu 2021A, Liu 2022, and a subset of Summers 2021 were excluded in accordance with our prespecified protocol. Cao 2021 included patients who were in CR at the start of therapy. Liu 2021A and Liu 2022 had a complicated study design involving sequential rounds of CAR T-cells where some patients only received CD19 CAR T-cells and not CD22 CAR-T cells, so efficacy outcomes were not comparable to the other included studies. Summers 2021 included two CAR T-cell products (V1 and V2), with a focus on the latter improved product, and did not clearly report CR among patients receiving V1. Out of 23 studies that included participants with B-ALL, 21 reported minimal residual disease (MRD) status.

**Figure 2:**
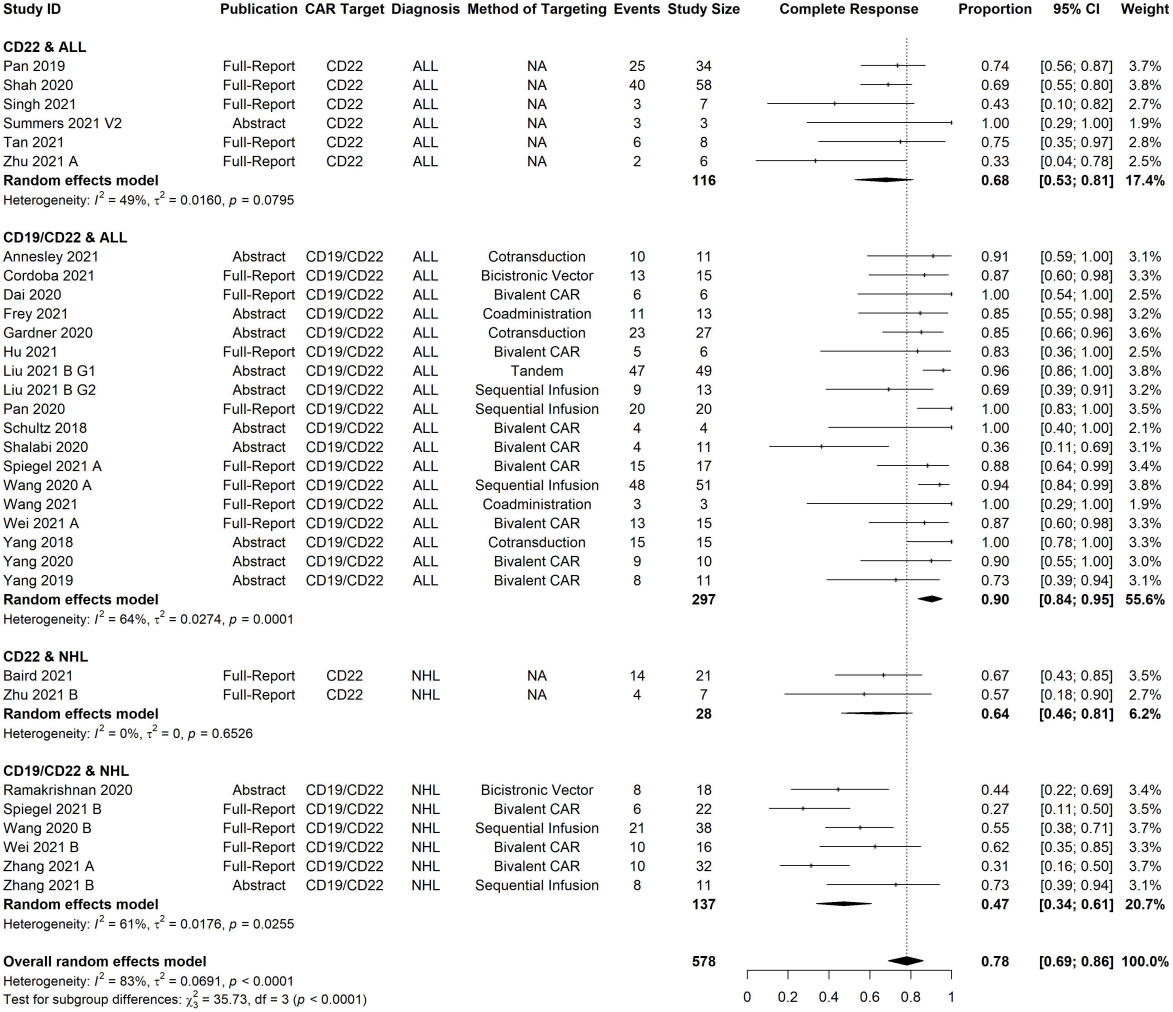
Forest plot of best complete response rate organized by malignancy type and antigen target. Pooled estimates, represented by the black diamond, were calculated for each subgroup and overall weighted effect.

The all-study model of bCR rate had significant heterogeneity (I^2 =^ 83%, p-value <0·0001). Through meta-regression with our pre-specified subgroups of interest, diagnosis (ALL vs. NHL) was identified as a significant predictor of bCR. (**Supplemental Table 2**). The residual heterogeneity was reduced to moderate (I^2 =^ 66%) by grouping studies according to both CAR target (CD22 vs CD19/CD22) and diagnosis (ALL vs. NHL). Therefore, we present the meta-analysis of bCR in these subgroups (**Figure 2**).

CD22 CAR T-cell therapies had an estimated CR of 68% [95% CI, 53-81%] in B-ALL patients (n=116), and 64% [95% CI, 46-81%] in NHL patients (n= 28). CD19/CD22 CAR T-cell therapies had an estimated CR rate of 90% [95% CI, 84-95%] in B-ALL patients (n= 297) and 47% [95% CI, 34-61%] in NHL patients (n= 137). The cumulative percentage of CRs which were MRD negative, among B-ALL studies which reported these data, was 93% (199/213) for CD19/CD22 CAR T-cells and 86% (68/79) for CD22 CAR T-cells.

Data for time-to-event outcomes, follow-up, relapse, and antigen status are presented in **Table 4**. Length of follow-up was reported in 20 studies, with reported median length ranging from 2-27.3 months. The methods of reporting relapse and survival were inconsistent with variable length of follow-up, limiting any direct comparisons. In general, relapse was common in these studies, with relapses being observed over a year after CR. Among CD22 studies that had at least 6 months median follow-up, the relapse rate range ranged from 17-69%, with Shah et al., 2020 having the longest median follow-up of 24 months and 49 out of 71 (69%) patients who achieved CR eventually relapsing. Among CD19/CD22 studies with at least 6 months of median follow-up, relapse rate ranged from 17-69%, with most of these studies having >40% relapse rates.

**Table 4:** Survival and Relapse Data from Included Studies.

Study ID	Disease	Months of follow up (median, range)	Relapse Rate (% of CR)	Overall survival data	Event-free survival
CD22 CAR T- cell Studies					
Pan 2019	ALL	3.2 (0-14.5)	6/26 (23%)	NR	1-year LFS rate (among those who achieved CR): 58.1%
Shah 2020	ALL	24*	49/71 (69%)	Median OS: 13.4 months (95% CI 7.7-20.3)	Median EFS: 3.2 months (95% CI 1.4-5.5) Median RFS (restricted to those in CR): 13.4 months (95% CI 7.7-20.3)
Singh 2021	ALL	IR	4/4 (100%)	NR	NR
Summers 2021 v2	ALL	NR	0/3 (0%)	NR	NR
Tan 2021	ALL	6 (2-11)	2/7 (29%)	IR	IR
Zhu 2021	ALL	IR	0/2 (0%)	6-month OS of pts who did not receive HSCT: 20.5%	6 month PFS of patients who did not receive HSCT: 20.0%
	DLBCL		0/4 (0%)	6-month OS of pts who did not receive HSCT: 67.07%	6 month PFS of patients who did not receive HSCT: 66.7%
Baird 2021	LBCL	7.3** (1.2-21.3)	3/18 (17%)	NR	NR
CD19/CD22 CAR T-cell Studies					
Cordoba 2021	ALL	14 (2-28)	9/13 (69%)	6 month OS rate: 80%	6 and 12-month EFS rate: 48%, 32% 6, 12-month molecular-free PFS rate: 38%, 23%
Dai 2020	ALL	8.5 (4-12)	3/6 (50%)	NR	NR
Frey 2021	ALL	6.2*** (0.2 - 25)	1/11 (9%)	6-month OS rate: 85%	NR
Gardner 2020	ALL	NR	4/23 (17%)	NR	NR
Hu 2021	ALL	4.3 (2-8)	1/5 (20%)	NR	NR
Liu 2021 A	ALL	19.7 (5.6-27.3)	After CD19: 3/26 After CD22: 5/19	ITT analysis of all 27 patients: 12-month OS rate: 84.0% (95% CI 70.7-99.8)	ITT analysis of all 27 patients: 12-month EFS rate: 65.2% (95% CI, 47.8 to 88.9)
Liu 2021 B	ALL	NR	NR	Tandem: 6-month OS rate: 90.0%	Tandem: 6-month LFS: 76.2%
				Sequential: 6-month OS rate: 88.9%	Sequential: 6-month LFS: 88.9%
Pan 2020	ALL	27.3 (9.8 to 36)	8/20 (40%)	2-year OS rate: 80.9% (95%CI 61.2-100.0%)	2-year LFS: 60% (95%CI, 38.5-81.5%)
Shalabi 2020	ALL	3.3 (1-8.5)	2/8 (25%)	NR	NR
Speigel 2021	ALL	9.3 (95% CI 7.2-NE)	10/17 (59%)	Median OS: 11.8 mo (95%CI 5.5-NE)	Median PFS: 5.8 months (95% CI 2.6–NE)
	LBCL	10 (95%CI 8.7 - 21.5)	8/13 (62%)	Median OS: 22.5 mo (95%CI 8.3–NE).	Median PFS: 3.2 months (95% CI 1.2–5.5)
Wang 2020	ALL	16.7 (1.3-33.3)	24/49 (49%)	Median OS: 31 months (95% CI 10.6-NR)	Median PFS: 13.6 months (95% CI, 6.5-NR); 1-year PFS rate: 52.9% (95% CI 38.5-65.5)
	NHL	14.4 (0.4-27.4)	NR	Median OS: 18 months (95% CI 6.1-NR)	Median PFS: 9.9 months (95% CI 3.3-NR); 1-year PFS rate: 50.0% (95% CI, 33.4-64.5)
Wang 2021	ALL	Incomplete reporting	2/4 (50%)	Incomplete reporting, only patient-level data	
Wei 2021	ALL	NR	3/4 (75%) of pts that did not proceed to HSCT	Median OS: 652 days (95% CI 390-905 days)	Median PFS: 90 days (95% CI 41-139 days)
	BCL	Median 397 days, range NR	7/14 (50%)	Median OS not reached 1-year OS rate: 77.3% 2-year OS rate: 77.3%	Median PFS: 246 days 1-year PFS rate: 40.2% 2-year PFS rate: 40.2%
Yang 2018	ALL	4.4 (0.8-13.1)	2/15 (13%)	NR	NR
Yang 2020	ALL	3.3 (0.5-7)	2/9 (22%)	Incomplete reporting, only patient-level data	
Yang 2019	ALL	2 (0.2-4.6)	0/8 (0%)	NR	NR
Cao 2021	NHL	24.3 (4.9 - 49.2)	N/A****	NR	NR
Liu 2022	NHL	17 (15-23)	After CD19: 6/15 (40%) After CD22: 0/13 (0%)	NR	18-month PFS: 78% (95% CI 55-90%)
Zhang 2021 A	NHL	8.7 (3-NR)	10/23 (43%)	Median OS not reached OS rate 69.1% at 6 months OS rate 63.3% at 12 months	Median PFS: 6.8 months PFS rate 51.4% at 6 months PFS rate 40.0% at 12 months
Zhang 2021 B	NHL	5.8 (3-NR)	0/8 (0%)	6-month OS rate: 100%	6-month PFS rate: 80.8%

LFS, leukemia-free survival; PFS, progression-free survival. Incomplete reporting includes studies that only reported follow-up for a subset of patients, or that include survival data in graphical form but do not provide numerical data points. *Shah et al. report “median potential follow-up” of 24 months. **Baird 2021 only reports median follow-up for patients in CR. ***Frey 2021 only reports median-follow-up for living patients. ****Cao 2021 included patients who were in CR at the start of remission, therefore is excluded from data involving CR rate. N/A, not applicable; NR, not reported.

Among CD22 CAR T-cell trials, Pan 2019 reported predominantly antigen-positive relapse while Shah 2020 reported predominantly antigen loss or diminished site density at relapse; Baird 2021 and Tan 2020 had small sample sizes with few relapses but reported 1/3 and 2/2 relapses involved CD22 loss or downregulation, respectively. Among CD19/CD22 CAR T-cell trials, the majority of relapses with reported antigen status were CD19+/CD22+ (42/50, 84%); among antigen-negative relapses, a common pattern observed was CD19-negative malignant cells with diminished CD22 site density (CD19-/CD22dim) (**Supplementary Materials**, p3).

*In-vivo* CAR T-cell expansion data was reported by 24 out of 30 studies. Both Wei 2021 and Hu 2021 found that patients who achieved CR had higher peak levels of CAR T-cells than non-responders. Long-term persistence of CAR T-cells was variable with limited reporting, and persistence ranged from 42 days to 10 months. Cao 2021 found that patients with progressive disease had no detectable CAR T-cells at three months, while most patients in CR did have detectable CAR T-cells at three months. Details of expansion and persistence data can be found in **Supplementary Materials** (page 3, **Supplementary Table 5**).

Data on manufacturing outcomes demonstrated no major challenges in CAR T-cell manufacturing, with bivalent CARs having comparable mean transduction efficacy to monovalent CARs. Details are presented in **Supplementary Materials** (page 3, **Supplementary Table 6**).

There was inadequate patient level data to perform subgroup analysis by age group, prior HSCT, prior CD19 CAR T-cell therapy, or other prior immunotherapies.

### Safety data

**Figure 3:**
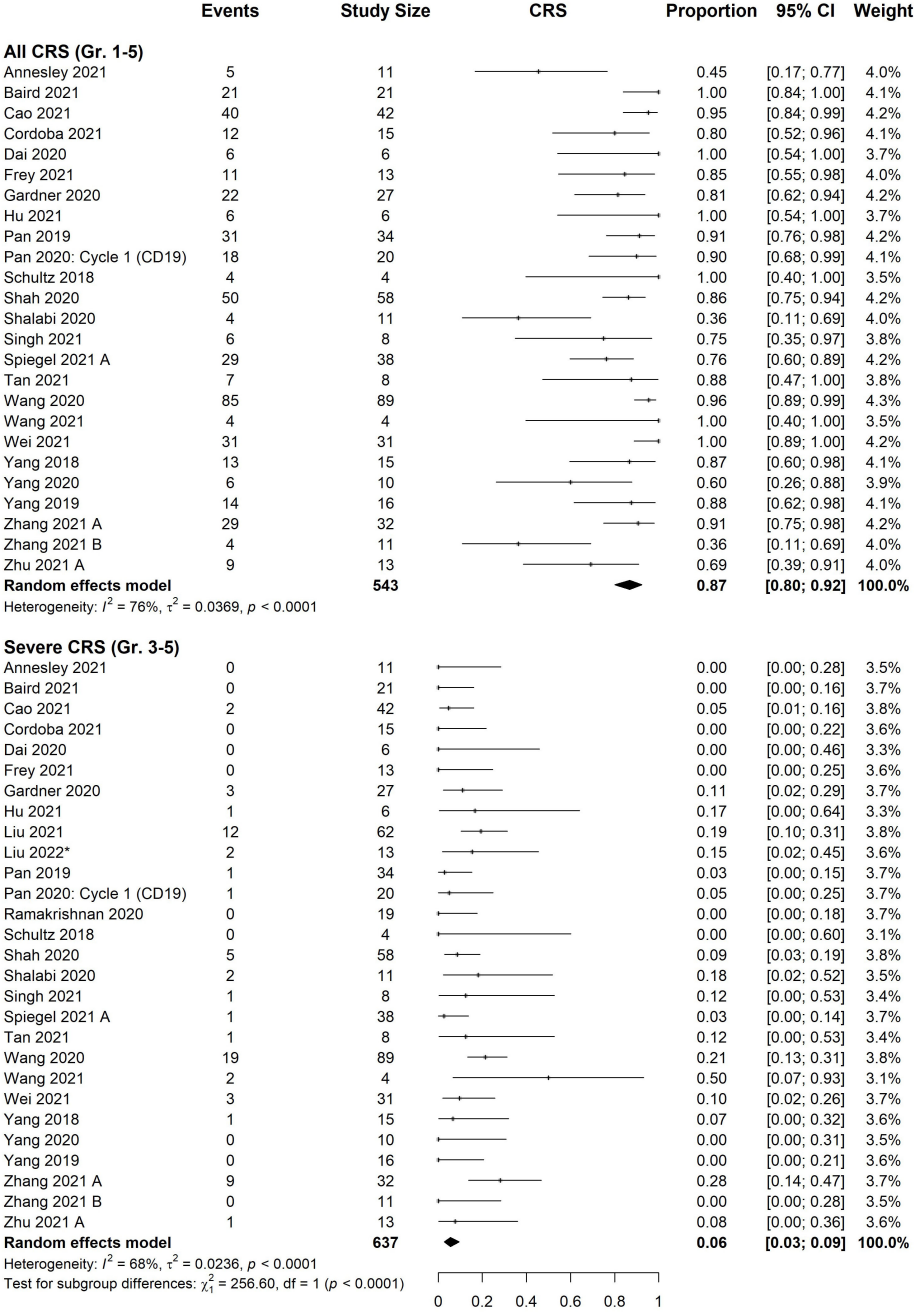
Forest plot of cytokine release syndrome rate organized by severity. Pooled estimate of effect, black diamond, was calculated for both all grades (1-5) and severe grades (3-5). Pan 2020: cycle 1 (CD19) was excluded from analysis, although rates did not significantly differ from cycle 2 (18/20 all grades, 1/20 severe).

**Figure 4:**
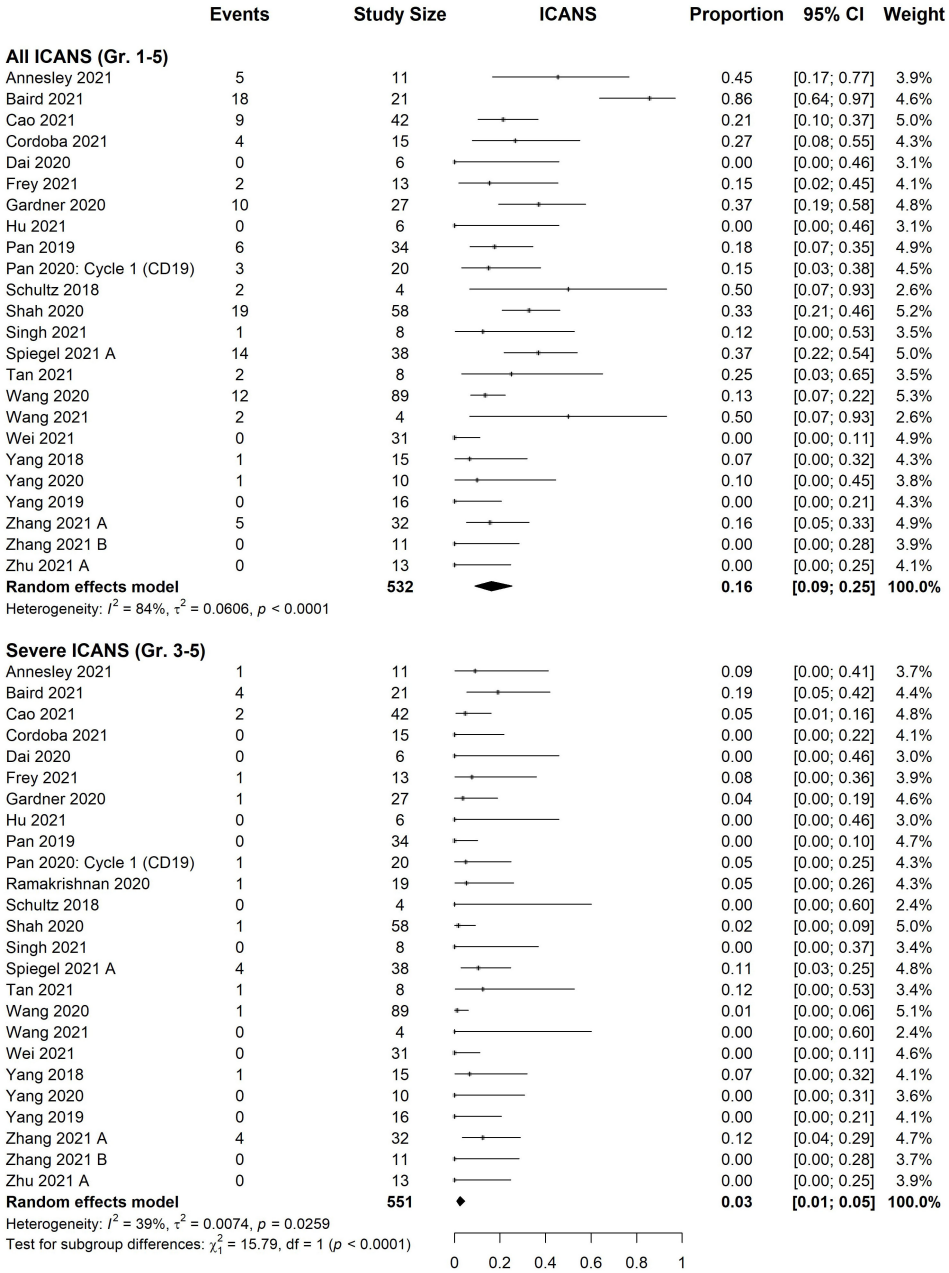
Forest plot of immune effector cell-associated neurotoxicity syndrome rate organized by severity. Pooled estimate of effect, black diamond, was calculated for both all grades (1-5) and severe grades (3-5). Pan 2020: cycle 1 (CD19) was excluded from analysis, although rates did not significantly differ from cycle 2 (3/20 all grades, 1/20 severe).

All studies provided information on CRS and ICANS. The estimated incidence of total and severe (grade ≥3) CRS were 87% [95% CI, 80%-92%] and 6% [95% CI, 3%-9%], respectively (**Figure 3**). Estimated ICANS and severe ICANS incidence were 16% [95% CI, 9-25%] and 3% [95% CI, 1-5%], respectively (**Figure 4**). Meta-regression revealed no significant difference in the incidence of adverse events (total or severe) between those treated with CD22 versus CD19/CD22 CAR T-cells (**Supplementary Table 3**). There was no significant difference in the incidence of CRS or ICANS (total or severe) between B-ALL and NHL patients.

All-cause 30-day mortality was available for 18 studies (n=457). The estimated incidence from these studies was 1% [95% CI, 0-3%] (**Supplementary Figure 1**). Pan 2019 and Frey 2021 were outliers with a 30-day mortality of 12% (4/34) and 15% (2/13), respectively. In Pan 2019, two patients died of infection-related causes, and two patients who had previously received transplantation had death attributable to a combination of CRS and graft reaction. In Frey 2021, one patient died of grade 4 ICANS and sepsis, and one died from a rapidly progressive disease.

Shah 2020 was the only study to report and characterize hemophagocytic histiocytosis (HLH)-like toxicities. In a retrospective analysis of 59 patients, they found that 21/52 (40%) of patients who developed CRS also developed HLH. The onset of HLH was delayed (median onset 14 days), and in 11/21 patients HLH developed after CRS was already resolving. HLH was effectively treated with corticosteroids and anakinra, however one patient died secondary to bacterial sepsis prior to HLH resolution. All other patients fully recovered.

### Bridging to hematopoietic cell transplant

Nineteen of the 30 studies commented on patients undergoing hematopoietic cell transplant (HCT) after achieving CR following CAR T-cell therapy. Among these, 99/292 (34%) of reported patients who achieved CR proceeding to HCT. Of note, this excludes Cao 2021, in which all patients received HCT prior to, rather than following, CAR T-cell therapy.

There was limited reporting of comparative survival in transplanted vs. non-transplanted groups. Shah 2020 found allogeneic HCT to be positively associated with relapse-free survival and event-free survival based on a time-covariate analysis. Similarly, Pan 2019 noted that at the observation endpoint, 8/11 CR patients who received transplant were relapse free (2 died of treatment-related mortality, 1 relapsed) while only 3/7 CR patients who had no further treatment were relapse free. In contrast, subgroup analysis of B-ALL patients by Wang 2020 found that transplant was not associated with a survival benefit.

Among studies examining HCT prior to CAR T-cell therapy, Cao 2021 showed significant efficacy of sequential HCT + CD19/CD22 CAR T-cells, with 2-year PFS of 83%. In Wang 2020, among the subset of patients that had a history of relapse post-HCT, 22/23 were able to achieve CR with CD19/CD22 CAR T-cell therapy, with 1-year PFS of 59.2%.

### Catalogue of registered clinical trials

Through our search of clinical trial databases, we catalogued 99 registered clinical trials investigating CAR T-cells that target CD22 alone (29%) or in combination with other antigen targets (63%). 34 of the 62 (54%) multi-targeting trials did not specify the multi-targeting approach used. 82 (83%) and 55 (56%) trial registries did not report the costimulatory domain(s) or T-cell source (allogeneic vs. autologous), respectively. Results have been reported for 36/99 (36%) of the registered trials so far, with three of these studies ineligible for inclusion in this review due to inadequate or absent reporting of clinical outcomes. Of note, we excluded 25 published reports which for which the corresponding clinical trial could not be identified and 33 publications that reported pooled outcomes of multiple clinical trials or CAR T-cell therapies, in which data on CD22-targeted therapies specifically could not be extracted (**Supplementary Materials**).

### Risk of bias

The risk of bias for all domains are presented in **Supplemental Figure 2** and summarized in **Supplemental Figure 3**. The majority of studies were single-center and did not provide estimates of random variability. No studies reported having blinded outcome assessors. The modified funnel plot appeared symmetrical, suggesting there was no publication bias (**Supplementary Figure 4**). The sensitivity analysis for publication type showed that removing data from conference abstracts did not substantially alter the estimates of CR (**Supplementary Figure 5**).

The evidence was assessed as low quality using the GRADE approach (**Supplementary Table 4**). While estimates were fairly consistent across studies, all studies were single-arm interventional studies with serious risks of bias.

## Discussion

We provide a narrative synthesis and meta-analysis of 30 early-phase single-arm studies representing 637 patients. There was a strong signal of efficacy with an estimated CR for CD22 CAR T-cells in B-ALL of 68% [95% CI, 54-77%], and 64% in NHL [95% CI, 46-81%]. Further, dual-targeting CD19/CD22 CAR T-cells had an estimated CR of 90% [95% CI, 84-98%] and 47% [95% CI, 34-61%] in B-ALL and NHL patients respectively. There was also an acceptable safety profile for both CD22 and CD19/CD22 CAR T-cells in R/R B-cell malignancies, with estimated rates of severe CRS and ICANS of just 6% [95% CI, 3%-9%] and 3% [95% CI, 1-5%].

Estimated CR rates in ALL patients were significantly higher with CD19/CD22 CAR T-cell therapy compared to single-target CD22 CAR T-cells. In contrast, the difference in bCR rates in NHL patients treated with CD19/CD22 CAR T-cells versus CD22 CAR T-cells was not statistically significant, however these groups did have a smaller sample size. It should be noted that a greater proportion of patients in the single-target CD22 CAR T-cell studies had failed CD19 CAR T-cells, and were receiving CD22 as a second-line CAR-T cell therapy. Thus, the lower CR rates seen among CD22 studies compared to dual target studies in ALL may be the result of selecting patients that were more refractory to treatment; in this case, substantial CR rates despite previous CAR T-cell failure point towards the value of CD22 CAR T-cells as a treatment option.

Prior meta-analyses of CD19 CAR T-cells reported estimated CR rates of 77% [95% CI, 63-87%] and 80% [95% CI, 76-85%] among ALL patients, which are similar to our estimated CR rates for CD22 CAR T-cells, but slightly lower than the CR rates estimated for CD19/CD22 CAR-T cells in ALL in our study.^4,8^ Among NHL patients, meta-analyses of CD19 CAR T-cells reported CR rates of 48% [95%CI: 42–54%] and 44% [95% CI: 34-55%], similar to our estimated CR for both CD22 CAR T-cells and CD19/CD22 CAR T-cells in NHL patients (26). This may indicate that dual therapy is more effective in ALL but not NHL patients, although direct comparison is not possible due to differing methodologies between meta-analyses, and given that prior meta-analyses included earlier generations of CAR T-cells that had lower efficacy. Among CD19/CD22 CAR T-cell therapies, a number of multi-targeting strategies were employed but given the small number of trials per group no comparison of efficacy could be made.

We saw no indication that dual-target CD19/CD22 therapies have a higher incidence of adverse events compared to single-target CD22 CAR T-cells. Compared to prior CD19 CAR T-cell meta-analyses, both CD22 and CD19/CD22 CAR T-cells had lower estimated rates of severe CRS and ICANS but higher rates of total CRS (5, 24). Notably, a retrospective study that compared CD19 and CD19/CD22 CAR T-cells from two clinical trials also showed that CD19 CAR T-cells actually had a statistically significant increased risk of severe CRS compared to CD19/CD22 CAR T-cells (80). These differences may be due to the inherent biology of the CARs or could be explained by variability in reporting guidelines, patients, dosing regimens, or improvement in treatment protocols. Overall 30-day mortality for all CD22 CAR T-cells was comparable to that of CD19 CAR T-cells and HCT (5, 81). In contrast to CD19 CAR T-cell studies, the incidence of adverse events was relatively homogenous across studies in our review (5).

Among studies that report long-term data, it appears that relapse within a year is common, and thus durability of response remains a challenge. However, there is inadequate data to determine whether relapse rates are significantly different to that of other CAR T-cell therapies, particularly given that many prior CD19 CAR T-cell studies used first-generation CARs, limiting the comparison to the current second-generation CD22 CARs. Among dual-target studies, relapses were often CD19+/CD22+, indicating that mechanisms other than antigen loss, including poor CAR T-cell persistence, may impact long-term outcomes. Long-term data are needed to determine whether dual-targeting improves relapse-free survival compared to single-targeting. Regardless, our study showed that CD22 CAR T-cells present another line of therapy for patients who relapse post-CAR T-cell therapy with CD19-negative disease.

Strengths of this review include our broad and methodical search strategy, which included both electronic searches of multiple databases and manual searching of conference abstracts. Sensitivity analysis confirmed that conference abstracts, although they had limited data on secondary outcomes, did not affect the heterogeneity of our meta-analysis, making them a valuable inclusion to our analysis. To further our confidence that all relevant research had been identified we searched clinical trial registries and catalogued our results.

Overall, many clinical trial registry entries in ClinicalTrials.gov or the WHO ICTRP poorly describe their respective intervention. Basic characteristics such as CAR target, structure, dosing regimen, and population criteria are often incomplete or missing. CAR T-cells are highly modular therapies and pre-clinical studies have shown that simple modifications (e.g. switching co-stimulatory domain, altering linker length) can have drastic effects on function (82–84). We strongly advocate for authors to follow a more robust and transparent approach to CAR T-cell trial registration, which should at a minimum provide the interventional and patient characteristics described in our protocol (30).

This review has a number of important limitations which should be recognized. Firstly, all studies are early-phase, single-arm, interventional trials, with a significant risk of bias. Concurrently, many outcomes of interest were not provided by the majority of studies. Nonetheless, there was sufficient data on CR rates and adverse events to achieve the primary aim of this review, which was to evaluate the efficacy and safety of CD22 CAR T-cell therapies in B-cell malignancies.

This systematic review of CD22 CAR-T cell clinical trials observed a strong signal of efficacy and safety of both single (CD22) and dual-target (CD19/CD22) CAR T-cells in patients with R/R B-cell malignancies. CD22 appears to be a viable antigen target and may be an option for those who relapse after CD19 CAR T-cell therapy. However, many early-phase interventional studies are still ongoing and have not yet published results, while others have reported preliminary results without having reached the maximum tolerated dose. The long-term efficacy of these therapies at their optimal therapeutic level thus has yet to be seen. Durability of response appears to remain a key limitation, as with other CAR T-cell therapies. Future trials are needed to determine the comparative efficacy of these therapies and identify strategies to improve the durability of response.

## Data availability statement

The original contributions presented in the study are included in the article/**Supplementary Material**. Further inquiries can be directed to the corresponding author.

## Author contributions

NF and KA (contributed equally): Literature search, compiled and verified the data, performed the statistical analysis and drafted manuscript. NK, HA and KH: Conceptualization, initial design, and supervision. All authors contributed to the article and approved the submitted version.
