# Supplementary material for: A systematic review and meta-analysis of CD22 CAR T-cells alone or in combination with CD19 CAR T-cells
Source: Front Immunol. 2023 Apr 27;14:1178403. doi: 10.3389/fimmu.2023.1178403 (PMC10174241; doi:10.3389/fimmu.2023.1178403)
Supplement: Supplementary file 1 [file DataSheet_1.pdf]

Supplementary Materials

Contents

Supplemental Methods 2

Search Strategy 2

Supplemental Results 3

Expansion and persistence data 3

Manufacturing outcomes 3

Adverse Events Grading Criteria 4

Supplemental Tables 5

Supplemental Table 1. Summary of Case Reports and Case Series 5

Supplemental Table 2. Meta-regression best CR 8

Supplemental Table 3. Meta-regression CRS and ICANS 9

Supplemental Table 4. Grade recommendations 10

Supplemental Table 5. CAR T-cell In-vivo Expansion and Persistence 11

Supplemental Table 6. CAR-T Cell Manufacturing Process and Outcomes 13

Supplemental Table 7. Antigen Status at Time of Relapse 16

Supplementary Figures 19

Supplemental Figure 1. All cause 30-day mortality. 20

Supplemental Figure 2. ROB Summary Table 21

Supplemental Figure 3. ROB Summary Graph 25

Supplemental Figure 4. Funnel plot of Arcsine transformed proportions of best CR vs study size. 26

Supplemental Figure 5. Sensitivity analysis for publication type on complete response incidence. Reproduction of Figure 1 but data from conference abstracts are censored in the meta-analysis. 28

Supplemental Figure 6. Overall response incidence organized by disease type and antigen target. 30

PRISMA Checklist 31

Included Studies and Associated Publications 35

Studies Excluded at Full-Text Screening Stage 41

Supplemental Methods

Search Strategy

Search Strategy for MEDLINE

1. ((chimeric antigen adj2 receptor*) and (therap* or treat* or immunity or immunotherap* or cell*)).tw,kw.

2. ((car adj3 t adj5 therap*) or (car adj3 t adj5 treat*)).tw,kw.

3. (car adj3 t adj3 immunotherap*).tw,kw.

4. Receptors, Antigen, T-Cell/tu

5. (car therap* or (car adj2 t adj2 cell*)).tw,kw.

6. ((modified or engineered) adj2 (t cell* or t lymphocyte*)).tw,kw.

7. Receptors, Antigen, T-Cell/ and (Adoptive Transfer/ or Immunotherapy, Adoptive/ or Immunotherapy/)

8. car t.tw,kw.

9. or/1-8

10. (cluster adj3 different* adj3 "22").tw.

11. (cd adj3 "22").tw.

12. (cd and "22").kf.

13. CAR22.mp.

14. CART-22.mp.

15. (CD19* and "22").mp.

16. cd22*.mp.

17. (CD20* and "22").mp.

18. 10 or 11 or 12 or 13 or 14 or 15 or 16 or 17

19. 9 and 18

20. 19 use medal

Supplemental Results

Expansion and persistence data

CAR T-cell in-vivo expansion and persistence was reported using different methods, with some studies reporting percent of total CD3+ T cells and others reporting copy number per volume of genomic DNA, limiting direct comparison.

In-vivo expansion was found to have a potential relationship with both CR and toxicity. Hu et al. 2021 found that patients who achieved CR/CRi had a greater peak expansion of CAR T-cells compared to non-responding patients (peak absolute cell counts 166.21 vs. 0.70 cells/mL; no comment on statistical significance). Wei et al. 2021 similarly found that peak expansion was 45.5% in patients who achieved CR, and 34.4% among those who did not ($p = 0.336$).

Spiegel et al. 2021 found that higher expansion was associated with increased CRS and neurotoxicity; they also saw CD8+ predominance over CD4+ during expansion despite predominance of CD4+ cells in the manufactured product.

Among CD19/CD22 studies involving co-transduction of two separate vectors for CD19 and CD22, Yang 2018 and Gardner 2020 both had greater expansion of CD19 CAR-expressing T-cells compared to CD22; conversely, in Annesley 2021, in-vivo expansion was predominated by CAR T-cells only expressing CD22, despite manufactured product containing both anti-CD22 and anti-CD19/CD22 cells.

Prior CAR T-cell therapy was found to potentially impact expansion. In Singh et al. 2021's study of CD22 single-target CAR T-cells, they found that patients with a history of CD19 CAR T-cell therapy actually had re-expansion of CD19 CARs when treated with CD22 CAR T-cells. Conversely, in Annesley et al. 2021's study, where in-vivo expansion was predominated by single CD22-expressing CAR T-cells and no CD19-expressing CARs, it was found that lack of CD19 CAR expression was most pronounced in patients with a history of prior CD19 CAR T-cell therapy.

Impact of CAR design on in-vivo expansion was explored by Summers et al. 2021. Their redesigned anti-CD22 CAR (V2) with a shorter linker and hinge, and addition of CD28 transmembrane domain, was found to have significantly greater in-vivo expansion than their original CAR design (V1), with expansion increasing over tenfold.

Eleven studies provided information on long-term CAR T-cell persistence. Reported median persistence ranged from 42 days to 10 months, with Wang 2020 having the longest reported persistence following infusion of sequentially infused CD19 and CD22 CAR T-cells in ALL and NHL patients, with comparable median persistence of CD19 versus CD22 CAR T-cells. Liu 2021A and Cao 2021, which also involved sequential infusion of CD19 and CD22 CAR T-cells, had lower rate of CD22 CAR T-cell persistence compared to CD19 CAR T-cell persistence (25% vs. 52% at 2 months in Liu 2021A; 69% vs. 97% at 3 months in Cao 2021). In Cao et al. 2021's study, patients who developed progressive disease had no detectable CAR T-cells at 2 or 3 months, while the majority of patients in CR had detectable CAR transgenes at 3 months.

Manufacturing outcomes

Studies that involved the manufacturing of single-target CAR T-cells (five CD22 single-target studies and three sequential infusion studies) had a mean CD22 CAR transduction efficacy of around 40% (25.8%-43.3%). Three Studies that involved manufacturing bivalent CARs had a transduction efficacy of around 50-60%, however, Dai 2020 had a lower transduction efficacy of 14%. Both studies involving bicistronic vector AUTO3 also had lower transduction efficacies of 17-18% (Supplemental Table 6). Annesley 2021 was the only study to involve co-transduction of two vectors that reported manufacturing outcomes. They had re-engineered their CAR T-cell product to enhance CD22-targeting, and the manufactured product had a mix of CD19/CD22 CAR T-cells (33%) and CD22-only CAR T-cells (42%), with few cells expressing solely the anti-CD19 CAR.

Adverse Events Grading Criteria

Only 20/29 studies included in the safety meta-analysis reported the criteria used to grade CRS. The most commonly used was the ASTCT criteria (n=8) followed by the Lee *et al.* criteria (n=7) and the University of Pennsylvania criteria (n=2), and others (CTCAE, NCCN 2019 criteria, and ASBMT consensus). Shah 2020 and Speigel 2021 both originally graded with the Lee criteria but retrospectively graded with the updated ASTCT consensus criteria. For ICANS, 16/29 studies reported the criteria used for grading (CTCAE criteria n=8, ASTCT criteria n=5, NCCN guidelines n=1, ASBMT critera n=1, author's own criteria n=1). Notably while the term "ICANS" used in this review reflects the new ASTCT grading criteria, many reports used term "neurotoxicity" and were published prior to the new criteria development.

Supplemental Tables

Supplemental Table 1. Summary of Case Reports and Case Series

Study ID	Age	Sex	Malignancy Description	CAR T-Cell Therapy	Outcome	Survival	Adverse Events
Lai 2017	31	M	R/R advanced stage IV FL	CD19 and CD22-targeted CAR T-cell therapy, method of co-targeting unclear	Achieved CR at 5 months	Continued CR at 6 months	Mild CRS (grade 0-1)
Li 2019A	46	M	Refractory DLBCL	CD19 and CD22-targeted CAR T-cell therapy, method of co-targeting unclear	Achieved "remission"	Remained in remission for 10 months	Grade 1 CRS
Li 20199B							
Meijing 2019	NR	F	TP53-positive refractory B-ALL	CD22 CAR-T cell therapy	CR	Continued CR 8 months	NR
Zhang 2019 Pt#1	6	NR	Burkitt's Lymphoma	Sequential administration of CD19 and CD22 CAR T-cells	PR after CD19, CR after CD22	Continued CR at 306 days	Grade 3 CRS
Zhang 2019 Pt#2	9	NR	Burkitt's Lymphoma	Sequential Administration of anti-CD19, CD22, and CD20 CAR T-cells	PR after CD19, PR after CD22, CR after CD20	Sustained remission for 128 days to date	Grade 3 CRS
Fu 2020	26	M	Ph-like ALL	Sequential administration of CD19 and CD22 CAR T-cells on successive days	CR	NR	NR
Hua 2020	10	F	Ph-like ALL, fourth relapse. Previous CD19 CAR-T and allo-HSCT.	Sequential Administration of donor-derived CD19 and CD22 CAR T-cell therapy	CR	Relapsed at 6 months after treatment	Grade 1 CRS
Jin 2020	44	M	Refractory ALL with FLT3-ITD mutations	Sequential Administration of CD19 and CD22 CAR T-cell therapy	MRD-negative CR	Continued CR for 3 months, bridged to haplo-HSCT, with sustained remission at 10 months follow up	Grade 2 CRS
Liang 2020	F	26	R/R B-ALL	“CD20/CD22 bispecific CART cells”	MRD-negative CR at two weeks	Sustained CR at 2 months	Gr. 3 CRS<br>No neurotoxicity
Wei 2020	M	26	Philadelphia chromosome-like ALL	Sequential infusion of CD19 and CD22 CAR T-cells	MRD-negative at day 22<br>allo-HSCT on day 64	Disease free for >6 months after allo-HSCT	CRS treated with ruxolitinib, and grade 1 CRES

Jain 2021*	NR	NR	Case series: 8 patients with R/R B-ALL	Group 1: UCART22 (allogeneically engineered CD22 CAR T-cells) (n=8); two dose levels evaluated (DL1 and DL2)<br>Group 2: UCART + alemtuzumab (n=3)	Group 2: All 3 patients had host lymphocyte suppression at 28 days	NR	Among group 2:<br>Gr.1 CRS (n=1)<br>Gr.3 hyperbilirubinemia and febrile neutropenia (n=1)<br>Gr. 1 pyrexia (n=1)<br>Infection (n=4)<br>No ICANS or GVHD
Jiao 2021** Pt.1	NR	32	R/R B-cell lymphoma (PMBCL)	4SCAR2.0 (4th generation multi-target CAR-T cell therapy):<br>Day 0: CD19/CD30 CAR T-cells<br>Day 42: CD19/CD22 CAR T-cells<br>Day 69: CD19-153Z CAR T-cells<br>Day 265: PSMA CAR T-cells	PR at day 26	CR at day 186<br>Continued CR at day 725	Grade 1 CRS<br>No neurotoxicity
Jiao 2021 Pt.3	NR	48	R/R B-cell lymphoma (FL/DLBCL)	4SCAR2.0:<br>Day 0: CD19/CD22 CAR T-cells<br>Day 7: GD2 CAR T-cells<br>Day 14: CD19-153Z CAR T-cells	PR after initial infusion<br>CR at day 22	Continued CR at day 453	No CRS or neurotoxicity
Jiao 2021 Pt.4	NR	61	R/R B-cell lymphoma (FL)	4SCAR2.0:<br>Day 0: CD19/CD22 CAR T-cells<br>D13: CD19-153z + CD22-153z CAR T-cells	PR after initial infusion<br>CR at day 49	Continued CR at day 290	No CRS or neurotoxicity
Sun 2021	M	39	B-ALL with extramedullary relapse after allogeneic stem cell transplantation, refractory to chemotherapy and radiotherapy	Received 4 courses of CAR T-cell therapy:<br>1. CD19 CART at month 46 post-HSCT<br>2. CD22 CART at month 54 post-HSCT<br>3. CD19 CART at month 57 post-HSCT<br>4. CD22 CART at month 63 post-HSCT	After 1st CD19: CR, then relapse at 4 months (CD19+CD22+)<br>After 1st CD22: MRD-neg CR in BM with decreased EM disease, then relapse at >1 month (CD19+CD22+)<br>After 2nd CD19: MRD-negative CRi in BM and EMD resolution at 3 months. At 5 months, CD19−CD22+ relapse.<br>After 2nd CD22: Transient decrease in blasts, however PD by day 28	No remission at 3 months	NR
Yan 2021 Pt#1	M	30	Refractory B-ALL, with subsequent EBV-associated post-transplant lymphoproliferative disorder (EBV-PTLD)	Two rounds of CAR T-cell treatment, both involving sequential infusion of CD22 and CD19 CAR T-cells on consecutive days	After 1st round of CD19+CD22: MRD-negative on day 28. Underwent allo-HSCT. After >1 month developed EBV-PTLD.<br>2nd round of CD19+CD22 CARTs given to treat EBV-PTLD, successfully resolved.	Passed away at 300 days post-HSCT due to multidrug-resistant pneumonia	Round 1: Gr. 3 CRS<br>Round 2: Gr. 1 CRS, Gr. 2 acute GVHD, Gr. 4 neutropenia and thrombocytoepenia

Yan 2021 Pt#2	F	10	R/R B-ALL with subsequent EBV-PTLD	Two rounds of CAR T-cell treatment, both involving sequential infusion of CD22 and CD19 CAR T-cells on consecutive days	After 1st round of CD19+CD22: CR followed by allo-HSCT, then developed EBV-PTLD.<br><br>2nd round of CD19+CD22 CARTs given to treat EBV-PTLD, successfully resolved.	EBV-DNA could not be detected up to 24 weeks of follow-up	Round 1: intermittent fever<br><br>Round 2: mild fever, grade 4 neutropenia+thrombocytopenia
Zi 2021	M	20	Refractory BCR-ABL(P210) positive B-ALL	"Bispecific" CD19/CD22 CAR T-cell therapy, method of co-targeting unclear	NR	NR	CRS refractory to tocilizumab and methylpredinosolone, treated with ruxolitinib

*Jain 2021 treated as case series due to small sample size and heterogenous intervention (3 patients received CAR T-cells alone at DL1 and 2 at DL2, and 3 patients received CAR T-cells+alemtuzumab). **Jiao 2021 patient #2 censored as this patient was in CR prior to intervention.

Supplemental Table 2. Meta-regression best CR

Predictor Variable	Estimate	SE	Z-Value	p-Value
**Intercept**	1.23	0.05	24.12	< 0.0001
**Diagnosis NHL (vs. ALL)**	-0.40	0.09	-4.55	< 0.0001
**CAR Target CD22 (vs. CD19/CD22)**	-0.17	0.09	-1.84	0.07

SE: Standard Error

Supplemental Table 3. Meta-regression CRS and ICANS

Model	Predictor Variable	Estimate	SE	Z-Value	p-Value
**CRS All**	Intercept	1.19	0.07	17.95	<0.0001
	Diagnosis NHL (vs. ALL)	-0.004	0.11	-0.03	0.97
	CAR Target CD22 (vs. CD19/CD22)	0.05	0.11	0.41	0.68
**CRS Severe**	Intercept	0.26	0.05	5.37	<0.0001
	Diagnosis NHL (vs. ALL)	-0.05	0.08	-0.62	0.53
	CAR Target CD22 (vs. CD19/CD22)	0.01	0.09	0.08	0.94
**ICANS All**	Intercept	0.36	0.07	5.14	<0.0001
	Diagnosis NHL (vs. ALL)	0.03	0.11	0.28	0.78
	CAR Target CD22 (vs. CD19/CD22)	0.12	0.12	0.98	0.33
**ICANS Severe**	Intercept	0.13	0.04	3.23	0.0012
	Diagnosis NHL (vs. ALL)	0.05	0.06	0.88	0.38
	CAR Target CD22 (vs. CD19/CD22)	0.001	0.07	0.02	0.99

SE: Standard Error

Supplemental Table 4. Grade recommendations

Outcome	Group	No. Studies	No. Patients	Domains					Estimate [95% CI]	Quality of Evidence
				ROB	Imprecision	Inconsistency	Indirectness	Publication Bias		
CR	CD22 ALL	6	116	Serious	Moderate	Non-serious	Non-serious	Non-Serious	0.68 [0.53-0.81]	Low
	CD19/CD22 ALL	18	297						0.90 [0.84-0.95]	
	CD22 NHL	2	28						0.64 [0.46-0.81]	
	CD19/CD22 NHL	8	137						0.47 [0.34-0.61]	
CRS	Total	30	543	Serious	Moderate	Non-serious	Non-serious	N/A	0.86 [0.80-0.91]	Low
	Severe								0.04 [0.01-0.08]	
ICANS	Total	30	532	Serious	Moderate	Non-serious	Non-serious	N/A	0.14 [0.08-0.22]	Low
	Severe								0.01 [0.00-0.03]	

Supplemental Table 5. CAR T-cell In-vivo Expansion and Persistence

Study ID	CAR T-cell Type	In-vivo Expansion (Median peak level of CAR T-cells)	CAR T-cell Persistence
Pan 2019	CD22	28.1% (0-86.5%) at days 12-15 (1.8-2200 fold).	NR
Shah 2020	CD22	77% at day 14-21 (peak was higher in those who underwent TCS than pre-TCS). Absolute value = 480.5 CAR T-cells/mL (39.7-11,346)	NR
Singh 2021	CD22	1977 (18-40314) copies/ug at day 11-18	NR
Summers 2021	CD22	V1: DL1: ~0.5%, DL2: ~0.9%<br>V2: ~12%	NR
Tan 2021	CD22	30% (1-45%), at days 11-15<br>Median count: 19.8 (range, 1.01–408) × 106/L	NR
Zhu 2021	CD22	2.24%, (2.04-9.28%) at 7-14 days<br>Median 240 (960-2580) copies/µg on day 14	NR
Baird 2021	CD22	85.4-350 cells/uL, peak at approximately 14 days<br>100- to 400-fold expansion	"Beyond 3 months"
Annesley 2021	CD19/CD22 cotransduction	~130 cells/uL, peak at 7-14 days	NR
Gardner 2020	CD19/CD22 cotransduction	CD19 CAR T-cells: 9.1%<br>CD22 CAR T-cells: 1.2%<br>CD19xCD22 CAR T-cells: 2.4%	NR
Yang 2018	CD19/CD22 cotransduction	CD19: 3.5 (0.47-79.1)x10^4 copies/mL PB genomic DNA<br>CD22: 0.9 (0.08-80.8)x10^4 copies/mL DNA on day 10 (7-14)	NR
Dai 2020	CD19/CD22 bivalent CAR	27% (12.8%-47.1%) at 2 weeks	NR
Hu 2021	CD19/CD22 bivalent CAR (universal CART)	CR/CRi patients:<br>- Peak 629,541copies/mg genomic DNA<br>- Peak absolute count: 166.21 (28.56-2072.37) cells/mL<br><br>Non-responding patients:<br>- Peak 27,347 copies/mg genomic DNA<br>- Peak absolute count: 0.70 cells/mL in non-responding patient	42 (21-114) days
Shalabi 2020	CD19/CD22 bivalent CAR	11.4 (0-84.22) cells/mL	43 (0-110) days (mean)
Spiegel 2021	CD19/CD22 bivalent CAR	36 cells/µl (interquartile range =13–136)<br>1,794 copies /50ng genomic DNA (IQR=509–4,315)	NR
Wei 2021	CD19/CD22 bivalent CAR	BCL: 40.6% (95% CI, 16.8%–54.9%)<br>B-ALL: 448 (63-4142.6) ×109 cells/uL	NR

Yang 2019	CD19/CD22 bivalent CAR	1.09 (0.0022-4.98) x 10^5 copy number/ug PB genomic DNA	NR
Yang 2020	CD19/CD22 bivalent CAR	2.29 $\times 10^5$ copies/µg genomic DNA (0.0014-5.66) peaking at day 14 (10-28)	Follow up to day 28 for 5 of 9 evaluable patients: Median 2.40 (0.75-3.98) $\times 10^5$ copies/µg genomic DNA
Zhang 2021 A	CD19/CD22 bivalent CAR	286294.4 copies/ug	92.5 days (range 13-763)
Cordoba 2021	CD19/CD22 bicistronic vector	46,717 copies per ug DNA	NR. Older report of this study (Amrolia et al. 2019) provided a median persistence of 180 (21-330) days.
Schultz 2018	CD19/CD22 bicistronic vector	10-25%	NR
Frey 2021	CD19/CD22 co-adminstration	NR<br>Median peak time:<br>CD19 CAR-T cells = 9 days<br>CD22 CAR T-cells = 16 days	Patients with detectable CAR T-cells at 3 months:<br>- 9/11 (82%) had detectable CD19 CARTs<br>- 8/11 (73%) had detectable CD22 CARTs<br><br>Patients with detectable CAR T-cells at 6 months:<br>- 7/8 (88%) had detectable CD19 CARTs<br>- 4/8 (50%) had detectable CD22 CARTs<br><br>All pts evaluable at 12 months had detectable CD19 and CD22 CAR T-cells
Cao 2021	CD19/CD22 sequential infusion (successive days)	Median peak expansion NR<br>CD19 CAR T-cells: peak at 7 (6-10) days<br>CD22 CAR T-cells: peak at 8 (6-14) days	Among 35 patients with ongoing CR, at 3 months:<br>- 34/35 (97%) had detectable CD19 CAR T-cells<br>- 24/35 (69%) had detectable CD22 CAR T-cells
Wang 2020	CD19/CD22 sequential infusion (successive days)	NR	ALL: CD19 = 10.7 months (0.5 - 33.3), CD22 = 10.0 months (0.2 - 16.7)<br><br>NHL: CD19 = 9.0 months (1.0 - 24.1), CD22 = 7.6 months (1.0 - 24.1)
Liu 2021 A	CD19/CD22 sequential infusion (long interval)	Median peak cell number:<br><br>- CD19: 6.92 (0-124) x 10^7/L<br>- CD22: 6.4 (0-128) x 10^7/L	At day 60 after CD19 CAR-T infusion:<br>- 14/27 (52%) had detectable CD19 CAR T-cells<br><br>At day 60 after CD22 CAR-T infusion:<br>- 5/20 (25%) had detectable CD22 CAR T-cells
Liu 2022	CD19/CD22 sequential infusion (long interval)	CD19: 7.45% (range, 0.00%–59.40%), peak at day 7<br>CD22: 41% (range, 0.22%–73.60%), peak at day 11<br>CD20: 2.97% (range, 0.60%–21.10%), peak at day 30	Median persistence of:<br>- CD19: 48 (30-101) days<br>- CD22: 49 (15-92) days<br>- CD20: 37.5 (15-140) days<br>50% of patients had detectable CAR T-cells at 6 months after first infusion (95% CI, 28%-69%)
Pan 2020	CD19/CD22 sequential infusion (long interval)	CD19: 9.84% (0.19-68.7); CD22: 23.9% (range: 0.3-85.2)	NR

Where peak expansion is reported as a percentage, this represents % of CD3+ cells. Liu 2021 B, Wang 2021, Ramakrishnan 2020, and Zhang 2021 B did not report any outcomes if interest.

Supplemental Table 6. CAR-T Cell Manufacturing Process and Outcomes

Study	Target	Vector	Method of dual-target	Manufacturing process details	Transduction efficacy
Pan 2019	CD22	LV	--	Before transduction: Leukapharesis products stimulated with anti-CD3/CD28 antibody-coated magnetic beads<br><br>After transduction: cultured in X-VIVO 15, a serum-free medium with 300 IU/ml IL-2	Mean: 41.3% (3.24%-70.8%)
Shah 2020	CD22	LV	--	Protocol was modified mid-trial to include CD4/CD8 T-cell selection (TCS), which was applied for 32/58 participants<br><br>Before transduction: Anti-CD3/CD28 stimulation, cultured in AIM V medium supplemented with 5% heat-inactivated human AB Serum, 1% Glutamax, and 40 IU/mL IL-2.<br><br>After transduction: cultured in medium with 100 IU/mL IL2	Mean: 40.7%<br><br>Mean transduction efficacy was significantly higher in TCS vs. pre-TCS (40.7% vs. 33.4% p=0.02)<br><br>1 product failure (pre-TCS).
Singh 2021	CD22	LV	--	Leukapheresis products stimulated with anti-CD3/CD28 paramagnetic beads	Pediatric: 36.4% (15%-49.7%)<br>Adult: 25.8% (25-30)<br>1 product failure (adult)
Tan 2021	CD22	LV	--	Leukapharesis products stimulated with magnetic beads coated with anti-CD3/CD28 antibodies.	Mean 43.2, median 41.65, range 27.7-57%<br>No product failures
Zhu 2021	CD22	NR	--	NR	Mean: 42.07% (±19.23%)<br>No product failures
Baird 2021	CD22	LV	--	Leukapharesis product enriched for CD4 and CD8 T-cells with TransAct before T-cell activation	NR<br>No product failures
Annesley 2021	CD19/CD22	LV	Cotransduction	Lekuapharesis product stimulated with anti-CD3/CD28 beads, and immunomagnetic selection of CD4/CD8 T cells. Compared two CAR-T products (V1 and V2), with aim to increase CD22 targeting.	V2 products had greater CD22 CAR expression than V1. Median expression in V2: 42% CD22 only, 33% CD19+CD22, 3.2% CD19 only.
Dai 2020	CD19/CD22	LV	Bivalent CAR	Before transduction: Anti-CD3/CD28 stimulation.<br><br>After transduction: cultured in AIM V medium with 5% human AB serum and 300 international units/mL IL-2.	Mean: 14.2% (10.32-16.91%)

Speigel 2021	CD19/CD22	LV	Bivalent CAR	Product was enriched for CD4 and CD8 T cells before T cell activation with TransAct.	Mean: 60.1% (range 34.6–75.2%)<br>Average vector copy number: 2.23<br>No product failures
Wei 2021	CD19/CD22		Bivalent CAR	Transduced cells cultured in X-VIVO 15, a serum-free medium with 300 IU/mL IL2, for duration of culture.	BCL patients: Median = 48.5% (22–70%)<br>B-ALL patients: Median = 60.1% (30-75.1%).
Zhang 2021 A	CD19/CD22	LV	Bivalent CAR	Leukapharesis product underwent isolation using anti-CD3 magnetic beads, followed by stimulation with monoclonal anti-CD3/CD28 antibodies.	NR<br>Leukapharesis failed in 1 patient
Hu 2021	CD19/CD22	LV	Bivalent CAR (CTA101)	Universal CAR-T cells made using CRISPR/Cas9 to disrupt TRAC region (to avoid host immune mediated CAR-T rejection) and CD52 gene (to allow for anti-CD52-mediated depletion of patients' T-cells).<br><br>Cells sorted via CliniMACS magnetic bead-mediated depletion of CD3 cells to minimize risk of GVHD	Approximate range 50-90% (estimated from figure)<br>Reported expansion above 100-fold<br>No product failures
Cordoba 2021	CD19/CD22	RV	Bicistronic vector (AUTO3)	T-cells expanded in Prodigy device	Median: 17.7% (range, 8.6–39.3%)<br>Median vector copy number: 0.55 (0.26–1.46).<br>1 product failure
Ramakrishnan 2020	CD19/CD22	RV	Bicistronic vector (AUTO3)	Products manufactured in semi-automated and closed process using CliniMACS Prodigy.	17% (range 16–29%)
Pan 2020	CD19/CD22	LV	Sequential infusion	Before transduction: stimulated with magnetic beads coated with anti-CD3/CD28 antibodies<br><br>After transduction: cultured in X-VIVO 15, a serum-free medium with 300 IU/ml IL-2	CD19: Mean = 48.7% (10.4-74.7%)<br><br>CD22: Mean = 42.8% (8.3-69.8%)
Cao 2021	CD19/CD22	LV	Sequential infusion	Product stimulated using Dynabeads CD3/CD28 T-cell activator in modified CTS OpTmizer T Cell Expansion serum-free medium at 37 °C and 5% CO2 for 18-24 hours	CD19: 38.51 +/- 15.22%<br>CD22: 43.32 +/- 14.25%<br>2 product failures (patients withdrawn from study)
Liu 2022	CD19/CD22	LV	Sequential infusion	Leukapharesis products stimulated with magnetic beads coated with anti-CD3 and anti-CD28 antibodies	NR<br>No product failures

Wang 2020	CD19/CD22	LV	Sequential infusion	Leukapharesed product underwent anti-CD3/CD28 stimulation in medium containing 2 mM l-glutamine, 5% human AB serum and 200IU/ml rhIL-2.	CD19: Mean = 40.4% (+/- 18.4%)<br>CD22: Mean = 42.8% (+/- 19.6%) and 3 product failures

LV: lentivirus. RV: retrovirus. The following included studies provided no relevant manufacturing outcomes: Summers 2021, Frey 2021, Gardner 2020, Liu 2021 A, Liu 2021 B, Schultz 2018, Shalabi 2020, Wang 2021, Yang 2018, Yang 2019, Yang 2020, Zhang 2021 B.

Supplemental Table 7. Antigen Status at Time of Relapse

Study ID	Baseline antigen status	Reporting of antigen status at relapse/progression
**CD22 CAR T-cell Studies**		
Pan 2019	All CD22-positive (>95% by flow cytometry)	- Leukemic cells of 4 out of 6 relapsing patients were examined to assess antigen status by flow cytometry<br>- All 4 had retained CD22 expression on leukemic cells and had no mutation or alternative splicing of CD22
Shah 2020	All CD22-positive	- Among 30 relapses:<br>- 7 participants had CD22-positive relapse<br>- 20 participants had relapse with CD22 loss or diminished site density<br>- 3 had unknown CD22 expression<br>- Median baseline (pre-intervention) CD22 site density higher in those that achieved MRD-positive CR (p = 0.02)
Singh 2021	All CD22-positive	- All 4 relapses were CD22-positive
Summers 2021	Not reported	- No reported relapses<br>- 3/4 patients with progressive disease were still CD22+ at day 21
Tan 2021	6 CD22-positive, 2 CD22-low	- Two relapses post-intervention: one was CD22-negative, and one had mixed CD22low and CD22-negative cells at relapse
Baird 2021	All CD22-positive	- CD22 expression downregulated/absent in 1 out of 3 patients evaluated at time of relapse
**CD19/CD22 CAR T-cell Studies**		
Cordoba 2021	Baseline CD19 and CD22 expression not reported for all patients	9 relapses, with antigen status reported for 3 patients:<br>- 1 CD19-/CD22- relapse (patient had mixed CD19-negative and CD19-positive disease at baseline)<br>- 1 CD19-negative relapse with reduced CD22 density<br>- 1 CD19-negative disease with unchanged CD22 density

Dai 2020		All CD19+/CD22+	Of the 3 relapses:<br>- 2 had no change in antigen expression at relapse<br>- 1 had CD19-negative cells at relapse with low but variable CD22 expression and diminished site density. Exon 2 deletion was seen in CD19 but no mutation in CD22.
Gardner 2020		"Diverse expression of CD19 and CD22" at baseline	Of the 4 relapses:<br>- 1 was CD19-/CD22<br>- 1 was CD19+/CD22+<br>- 2 were CD19-/CD22+
Pan 2020		All CD19+/CD22+ (>95% by flow cytometry)	Of the 3 relapses:<br>- 1 had CD22 downregulation<br>- 2 had CD19 antigen loss
Shalabi 2020		NR	2/2 relapses were CD19+/CD22+
Speigel 2021	LBCL	All CD19+ at baseline. Baseline CD22 expression was heterogenous (at least 3 pts were CD22-low).	Of subset of patients reassessed at relapse:<br>- 4 were CD19-negative or CD19-low<br>- 4 were CD22-negative (1 of whom had undetectable CD22 expression at baseline)<br>- 2 were CD22-low (1 was CD22-low at baseline)
	ALL	All CD19+.<br>Baseline CD22 status incompletely reported.	- At disease progression, 5/10 patients had CD19-negative or low expression with no change in CD22 expression
Wang 2020	NHL	ALL CD19+/CD22+	- 7 out of 18 patients who progressed were biopsied to assess antigen status<br>- All were CD19+/CD22+
	ALL	All CD19+/CD22+	- 23/24 relapses were CD19+/CD22+<br>- 1 relapse was CD19-neg/CD22-dim
Yang 2018		All CD19+/CD22+	2/2 relapses were CD19+/CD22+
Yang 2020		NR	2/2 relapses were CD19+/CD22+
Cao 2021		All CD19+/CD22+	No antigen loss observed at time of progression

Liu 2022	All CD19+ (4/23 had <30% expression)<br><br>All CD22+ (4/23 had <30% expression)	- Antigen status only reported post-CD19 CAR T-cell intervention:<br>- 4 patients biopsied, 3 had preserved CD19 expression and 1 was CD19-neg<br>- No antigen status reported post-CD22 CAR T-cell intervention

The reporting of relapse and antigen expression was highly variable between studies. Specifically, while some studies identified CD22 site density as a measure associated with relapse, other studies only report CD22 expression in binary terms (CD22-negative or positive).

Supplementary Figures

Study ID	CAR_Target	Diagnosis	Method of Targeting	Events	Study Size	Deaths Within 1 Month	Proportion	95% CI	Weight
Baird 2021	CD22	NHL	NA	1	21		0.05	[0.00; 0.24]	5.4%
Cao 2021	CD19/CD22	NHL	Sequential Infusion	0	42		0.00	[0.00; 0.08]	7.8%
Dai 2020	CD19/CD22	ALL	Bivalent CAR	0	6		0.00	[0.00; 0.46]	2.1%
Frey 2021	CD19/CD22	ALL	Coadministration	2	13		0.15	[0.02; 0.45]	3.9%
Hu 2021	CD19/CD22	ALL	Bivalent CAR	0	6		0.00	[0.00; 0.46]	2.1%
Pan 2019	CD22	ALL	NA	4	34		0.12	[0.03; 0.27]	7.0%
Pan 2020: Cycle 1 (CD19)	CD19/CD22	ALL	Sequential Infusion	0	20		0.00	[0.00; 0.17]	5.2%
Pan 2020: Cycle 2 (CD22)	-	-	-	0	20		0.00	[0.00; 0.17]	5.2%
Shah 2020	CD22	ALL	NA	1	58		0.02	[0.00; 0.09]	8.9%
Singh 2021	CD22	ALL	NA	0	8		0.00	[0.00; 0.37]	2.7%
Spiegel 2021 A	CD19/CD22	ALL & NHL	Bivalent CAR	0	38		0.00	[0.00; 0.09]	7.4%
Tan 2021	CD22	ALL	NA	0	8		0.00	[0.00; 0.37]	2.7%
Wang 2020	CD19/CD22	ALL & NHL	Sequential Infusion	3	89		0.03	[0.01; 0.10]	10.2%
Wang 2021	CD19/CD22	ALL	Coadministration	0	4		0.00	[0.00; 0.60]	1.5%
Wei 2021	CD19/CD22	ALL & NHL	Bivalent CAR	0	31		0.00	[0.00; 0.11]	6.7%
Yang 2018	CD19/CD22	ALL	Cotransduction	0	15		0.00	[0.00; 0.22]	4.3%
Yang 2020	CD19/CD22	ALL	Bivalent CAR	0	10		0.00	[0.00; 0.31]	3.2%
Yang 2019	CD19/CD22	ALL	Bivalent CAR	0	11		0.00	[0.00; 0.28]	3.4%
Zhang 2021 A	CD19/CD22	NHL	Bivalent CAR	3	32		0.09	[0.02; 0.25]	6.8%
Zhang 2021 B	CD19/CD22	NHL	Sequential Infusion	0	11		0.00	[0.00; 0.28]	3.4%
**Random effects model**					**477**		**0.01**	**[0.00; 0.03]**	**100.0%**

Heterogeneity: $I^2 = 40\%$, $\tau^2 = 0.0073$, $p = 0.0343$

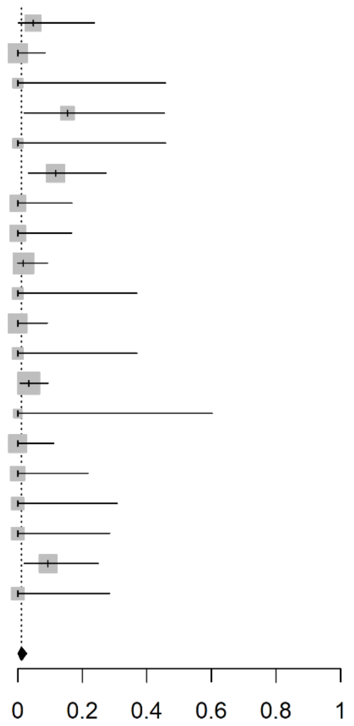

Supplemental Figure 1. All cause 30-day mortality.

	Did patients enter the study at a similar point in the disease?	Did the study provide estimates of random variability in the data analysis of relevant outcomes?	The study does not perform selective outcome reporting.	Was follow-up period reported?	Was the hypothesis/ aim/ objective of the study stated?	Was the intervention of interest described?	Was the study conducted prospectively?	Were additional interventions clearly described?	Were both competing interests and sources of support for the study reported?	Were details of the statistical tests reported?	Were outcome assessors blinded to the intervention that patients received?	Were patients from more than one centre?	Were patients recruited consecutively?	Were relevant outcome measures established a priori in the introduction or methods section?	Were the adverse events reported?	Were the characteristics of the patients included in the study described?	Were the eligibility criteria (i.e. inclusion and exclusion criteria) for entry into the study clearly stated?	Were the relevant outcome measures made before and after the intervention? for All outcomes	Were the relevant outcomes measured using appropriate objective or subjective methods?
Zhu 2021	low	low	low	low	low	unclear	low	low	low	low	high	high	unclear	low	low	low	unclear	low	low
Zhang 2021	low	low	low	low	low	high/low	low	unclear/low	unclear/low	low	high	high	unclear	unclear/low	low	unclear/high	unclear/low	low	low
Zhang 2019	low	low	unclear	low	low	unclear	low	unclear	unclear	unclear	high	high	unclear	unclear	unclear	unclear	unclear	low	unclear
Yang 2020	low	low	low	low	low	low	low	low	unclear	low	high	high	unclear	unclear	low	low	unclear	unclear	low
Yang 2019	low	high	unclear	low	low	unclear	low	unclear	low	unclear	high	high	unclear	unclear	unclear	low	low	low	unclear
Yang 2018	low	high	unclear	low	low	unclear	low	unclear	low	unclear	high	unclear	unclear	low	unclear	low	unclear	low	unclear
Wei 2021	low	low	low	low	low	low	unclear	low	low	low	high	high	unclear	low	low	low	low	low	low
Wang 2021	unclear	low	low	unclear	low	unclear	low	low	low	low	high	high	unclear	low	low	low	unclear	low	low
Wang 2020	low	low	low	low	low	low	low	low	low	low	high	high	unclear	low	low	low	low	low	low
Tan 2021	low	low	low	low	low	low	low	low	low	low	high	high	unclear	low	low	low	low	low	low
Summers 2021	low	low	unclear	high	unclear	unclear	low	low	low	unclear	high	unclear	unclear	unclear	high	high	high	low	unclear
Spiegel 2021	low	low	low	low	low	low	low	low	low	low	high	high	unclear	low	low	low	low	low	low
Singh 2021	high	low	low	unclear	low	low	low	low	low	low	high	unclear	unclear	low	low	unclear	low	low	low
Shalabi 2020	low	low	unclear	unclear	low	low	low	unclear	high	low	high	unclear	low	low	unclear	unclear	unclear	low	low
Shah 2020	low	low	low	low	low	low	low	low	low	low	high	high	unclear	low	low	unclear	low	low	low
Schultz 2018	low	low	unclear	high	low	low	low	low	low	low	high	high	unclear	unclear	unclear	high	unclear	low	low
Ramakrishnan 2020	low	high	low	high	low	unclear	low	low	unclear	unclear	high	low	unclear	low	unclear	unclear	low	unclear	unclear
Pan 2020	low	high	low	low	low	low	low	low	low	low	high	high	unclear	low	low	low	low	low	low
Pan 2019	low	low	unclear	low	low	low	low	low	low	low	high	high	unclear	low	unclear	low	low	low	low
Liu 2022	low	low	low	low	low	low	low	low	low	low	high	high	unclear	low	unclear	unclear	low	low	low
Liu 2021	low	high	unclear	high	low	high	low	unclear	unclear	unclear	high	high	unclear	unclear	high	high	unclear	low	low
Hu 2021	low	low	low	low	low	low	low	low	low	low	high	high	unclear	low	low	low	unclear	low	low
Gardner 2020	unclear	high	low	high	unclear	unclear	low	low	unclear	unclear	high	low	unclear	unclear	unclear	unclear	low	unclear	unclear
Frey 2021	low	low	low	low	low	unclear	low	unclear	low	low	high	high	unclear	low	low	unclear	unclear	low	low
Dai 2020	low	high	low	unclear	low	low	low	low	low	unclear	high	high	unclear	low	unclear	low	unclear	low	low
Cordoba 2021	low	low	low	low	low	low	low	low	low	low	high	low	unclear	low	low	low	low	low	low
Cao 2021	low	low	low	low	low	low	low	low	low	low	high	high	unclear	low	low	low	low	low	low
Baird 2021	low	low	low	low	low	low	low	low	low	low	high	high	unclear	low	low	low	low	low	low
Annesley 2021	unclear	high	low	high	unclear	low	low	unclear	low	unclear	high	unclear	unclear	low	unclear	high	unclear	low	low

Supplemental Figure 2. ROB Summary Table

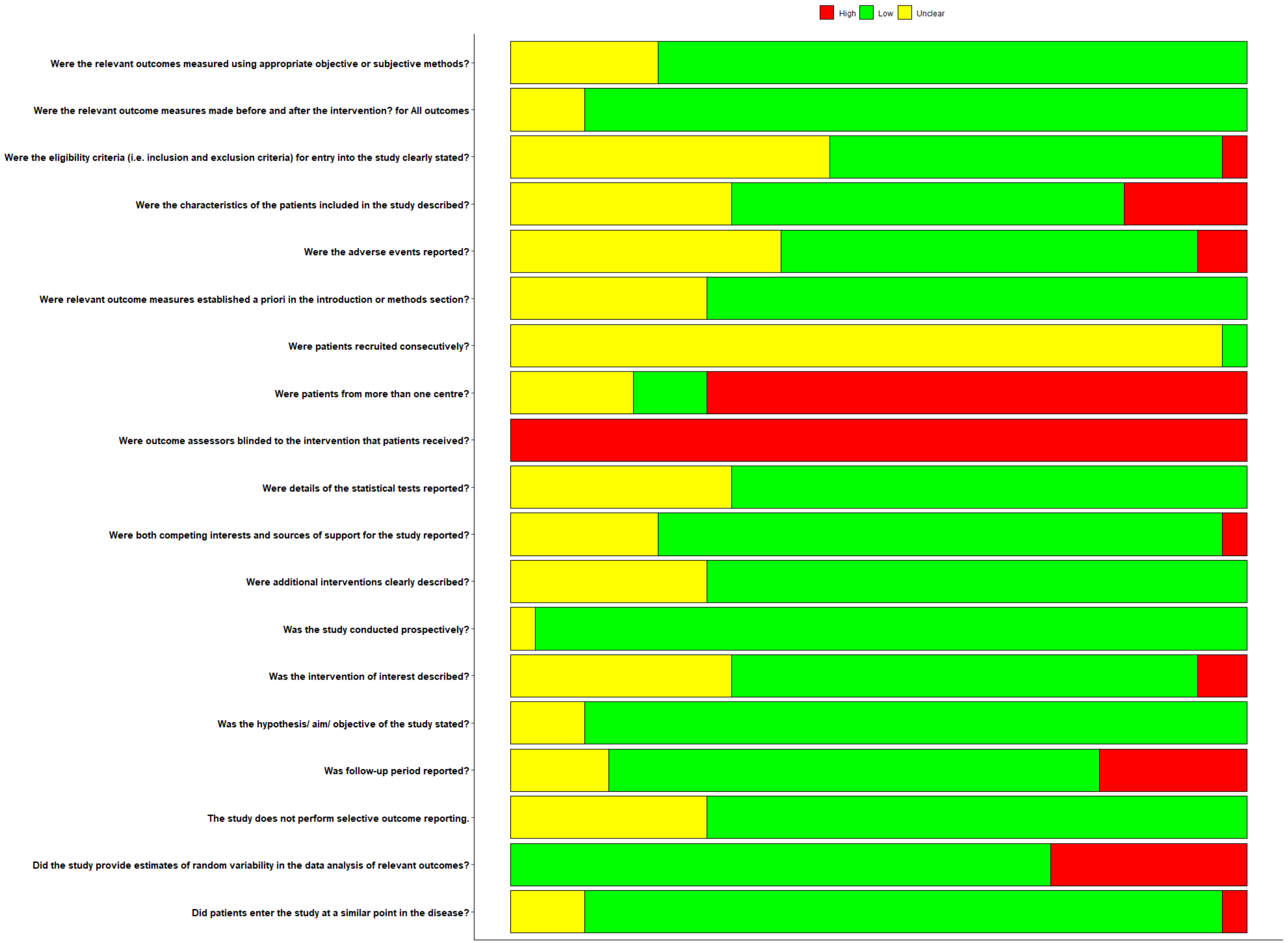
High
Low
Unclear
Were the relevant outcomes measured using appropriate objective or subjective methods?
Were the relevant outcome measures made before and after the intervention? for All outcomes
Were the eligibility criteria (i.e. inclusion and exclusion criteria) for entry into the study clearly stated?
Were the characteristics of the patients included in the study described?
Were the adverse events reported?
Were relevant outcome measures established a priori in the introduction or methods section?
Were patients recruited consecutively?
Were patients from more than one centre?
Were outcome assessors blinded to the intervention that patients received?
Were details of the statistical tests reported?
Were both competing interests and sources of support for the study reported?
Were additional interventions clearly described?
Was the study conducted prospectively?
Was the intervention of interest described?
Was the hypothesis/ aim/ objective of the study stated?
Was follow-up period reported?
The study does not perform selective outcome reporting.
Did the study provide estimates of random variability in the data analysis of relevant outcomes?
Did patients enter the study at a similar point in the disease?

Supplemental Figure 3. ROB Summary Graph

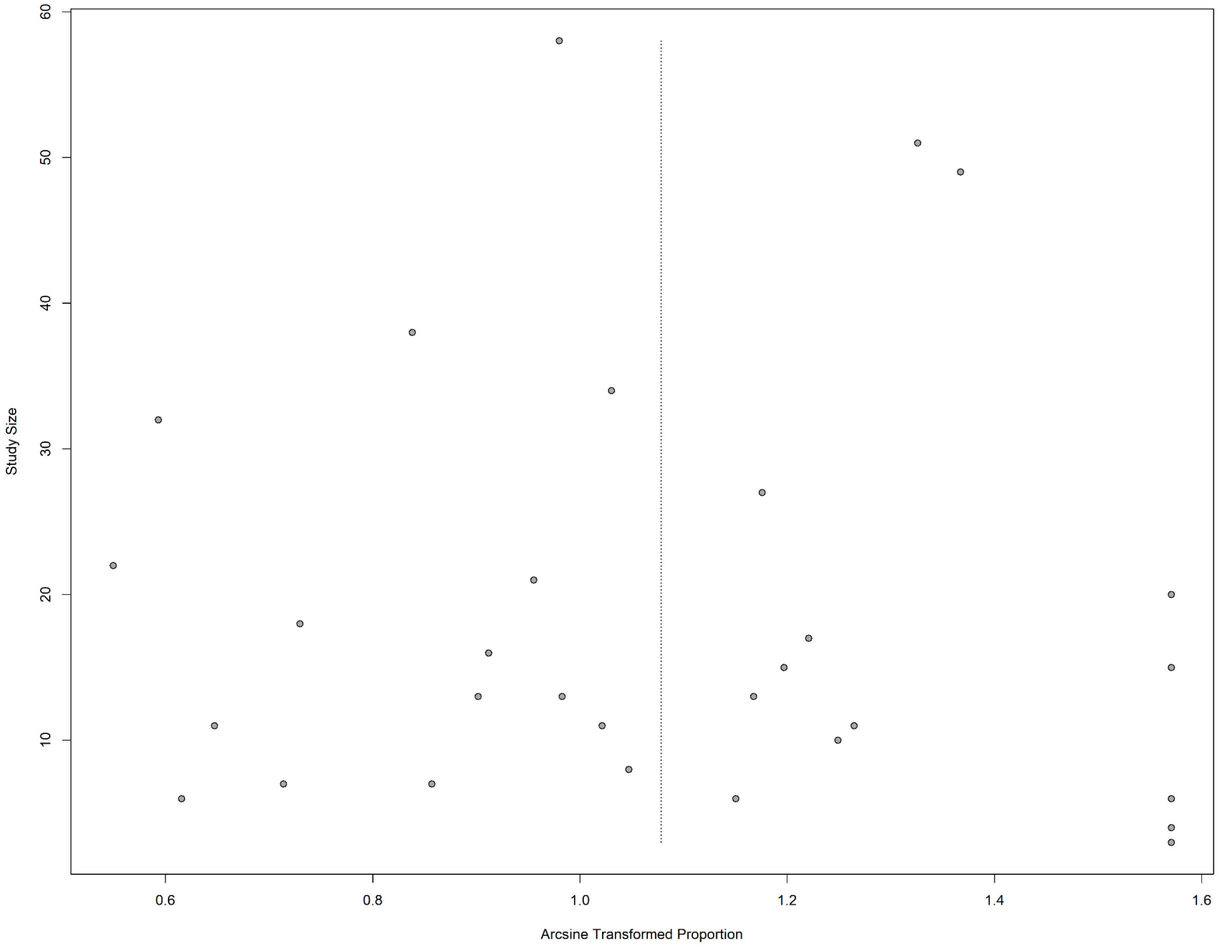

Supplemental Figure 4. Funnel plot of Arcsine transformed proportions of best CR vs study size.

Study ID	Publication	CAR Target	Diagnosis	Method of Targeting	Events	Study Size	Proportion	95% CI	Weight
**CD22 & ALL**									
Pan 2019	Full-Report	CD22	ALL	NA	25	34	0.74	[0.56; 0.87]	6.3%
Shah 2020	Full-Report	CD22	ALL	NA	40	58	0.69	[0.55; 0.80]	6.7%
Singh 2021	Full-Report	CD22	ALL	NA	3	7	0.43	[0.10; 0.82]	4.4%
Summers 2021 V2	Abstract	CD22	ALL	NA	3	3	1.00	[0.29; 1.00]	0.0%
Tan 2021	Full-Report	CD22	ALL	NA	6	8	0.75	[0.35; 0.97]	4.6%
Zhu 2021 A	Full-Report	CD22	ALL	NA	2	6	0.33	[0.04; 0.78]	4.1%
**Random effects model**						**116**	**0.66**	**[0.54; 0.77]**	**26.2%**
Heterogeneity: $I^2 = 28\%$, $\tau^2 = 0.0054$, $p = 0.2355$									
**CD19/CD22 & ALL**									
Annesley 2021	Abstract	CD19/CD22	ALL	Cotransduction	10	11	0.91	[0.59; 1.00]	0.0%
Cordoba 2021	Full-Report	CD19/CD22	ALL	Bicistronic Vector	13	15	0.87	[0.60; 0.98]	5.5%
Dai 2020	Full-Report	CD19/CD22	ALL	Bivalent CAR	6	6	1.00	[0.54; 1.00]	4.1%
Frey 2021	Abstract	CD19/CD22	ALL	Coadministration	11	13	0.85	[0.55; 0.98]	0.0%
Gardner 2020	Abstract	CD19/CD22	ALL	Cotransduction	23	27	0.85	[0.66; 0.96]	0.0%
Hu 2021	Full-Report	CD19/CD22	ALL	Bivalent CAR	5	6	0.83	[0.36; 1.00]	4.1%
Liu 2021 B G1	Abstract	CD19/CD22	ALL	Tandem	47	49	0.96	[0.86; 1.00]	0.0%
Liu 2021 B G2	Abstract	CD19/CD22	ALL	Sequential Infusion	9	13	0.69	[0.39; 0.91]	0.0%
Pan 2020	Full-Report	CD19/CD22	ALL	Sequential Infusion	20	20	1.00	[0.83; 1.00]	5.9%
Schultz 2018	Abstract	CD19/CD22	ALL	Bivalent CAR	4	4	1.00	[0.40; 1.00]	0.0%
Shalabi 2020	Abstract	CD19/CD22	ALL	Bivalent CAR	4	11	0.36	[0.11; 0.69]	0.0%
Spiegel 2021 A	Full-Report	CD19/CD22	ALL	Bivalent CAR	15	17	0.88	[0.64; 0.99]	5.7%
Wang 2020 A	Full-Report	CD19/CD22	ALL	Sequential Infusion	48	51	0.94	[0.84; 0.99]	6.6%
Wang 2021	Full-Report	CD19/CD22	ALL	Coadministration	3	3	1.00	[0.29; 1.00]	2.9%
Wei 2021 A	Full-Report	CD19/CD22	ALL	Bivalent CAR	13	15	0.87	[0.60; 0.98]	5.5%
Yang 2018	Abstract	CD19/CD22	ALL	Cotransduction	15	15	1.00	[0.78; 1.00]	0.0%
Yang 2020	Abstract	CD19/CD22	ALL	Bivalent CAR	9	10	0.90	[0.55; 1.00]	0.0%
Yang 2019	Abstract	CD19/CD22	ALL	Bivalent CAR	8	11	0.73	[0.39; 0.94]	0.0%
**Random effects model**						**297**	**0.94**	**[0.88; 0.98]**	**40.4%**
Heterogeneity: $I^2 = 33\%$, $\tau^2 = 0.0082$, $p = 0.1650$									
**CD22 & NHL**									
Baird 2021	Full-Report	CD22	NHL	NA	14	21	0.67	[0.43; 0.85]	5.9%
Zhu 2021 B	Full-Report	CD22	NHL	NA	4	7	0.57	[0.18; 0.90]	4.4%
**Random effects model**						**28**	**0.64**	**[0.46; 0.81]**	**10.3%**
Heterogeneity: $I^2 = 0\%$, $\tau^2 = 0$, $p = 0.6526$									
**CD19/CD22 & NHL**									
Ramakrishnan 2020	Abstract	CD19/CD22	NHL	Bicistronic Vector	8	18	0.44	[0.22; 0.69]	0.0%
Spiegel 2021 B	Full-Report	CD19/CD22	NHL	Bivalent CAR	6	22	0.27	[0.11; 0.50]	6.0%
Wang 2020 B	Full-Report	CD19/CD22	NHL	Sequential Infusion	21	38	0.55	[0.38; 0.71]	6.4%
Wei 2021 B	Full-Report	CD19/CD22	NHL	Bivalent CAR	10	16	0.62	[0.35; 0.85]	5.6%
Zhang 2021 A	Full-Report	CD19/CD22	NHL	Bivalent CAR	10	32	0.31	[0.16; 0.50]	0.0%
Zhang 2021 B	Abstract	CD19/CD22	NHL	Sequential Infusion	8	11	0.73	[0.39; 0.94]	5.1%
**Random effects model**						**137**	**0.53**	**[0.35; 0.71]**	**23.1%**
Heterogeneity: $I^2 = 66\%$, $\tau^2 = 0.0235$, $p = 0.0336$									
**Overall random effects model**						**578**	**0.76**	**[0.65; 0.86]**	**100.0%**
Heterogeneity: $I^2 = 81\%$, $\tau^2 = 0.0565$, $p < 0.0001$									
Test for subgroup differences: $\chi^2_3 = 33.13$, df = 3 ($p < 0.0001$)									

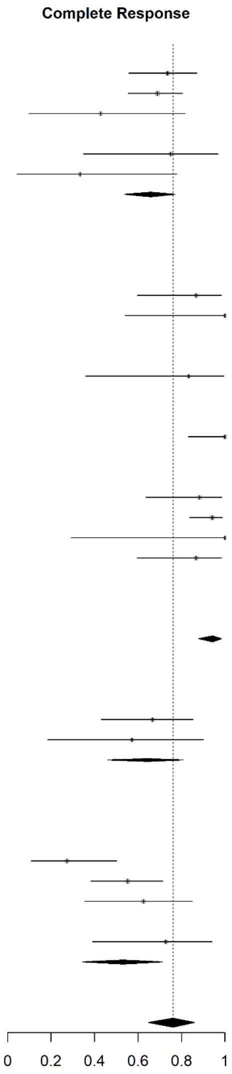

Supplemental Figure 5. Sensitivity analysis for publication type on complete response incidence. Reproduction of Figure 1 but data from conference abstracts are censored in the meta-analysis.

Study ID	Publication	CAR Target	Diagnosis	Method of Targeting	Events	Study Size	Proportion	95% CI	Weight
**CD22 & ALL**									
Pan 2019	Full-Report	CD22	ALL	NA	26	34	0.76	[0.59; 0.89]	4.8%
Shah 2020	Full-Report	CD22	ALL	NA	41	58	0.71	[0.57; 0.82]	5.1%
Singh 2021	Full-Report	CD22	ALL	NA	3	7	0.43	[0.10; 0.82]	3.0%
Summers 2021 V1	Abstract	CD22	ALL	NA	NA	4			0.0%
Summers 2021 V2	Abstract	CD22	ALL	NA	3	3	1.00	[0.29; 1.00]	1.8%
Tan 2021	Full-Report	CD22	ALL	NA	7	8	0.88	[0.47; 1.00]	3.2%
Zhu 2021 A	Full-Report	CD22	ALL	NA	3	6	0.50	[0.12; 0.88]	2.8%
**Random effects model**						**120**	**0.73**	**[0.59; 0.85]**	**20.8%**
Heterogeneity: $I^2 = 45\%$, $\tau^2 = 0.0136$, $p = 0.1676$									
**CD19/CD22 & ALL**									
Annesley 2021	Abstract	CD19/CD22	ALL	Cotransduction	NA	11			0.0%
Cordoba 2021	Full-Report	CD19/CD22	ALL	Bicistronic Vector	13	15	0.87	[0.60; 0.98]	4.0%
Dai 2020	Full-Report	CD19/CD22	ALL	Bivalent CAR	6	6	1.00	[0.54; 1.00]	2.8%
Frey 2021	Abstract	CD19/CD22	ALL	Coadministration	11	13	0.85	[0.55; 0.98]	3.8%
Gardner 2020	Abstract	CD19/CD22	ALL	Cotransduction	NA	27			0.0%
Hu 2021	Full-Report	CD19/CD22	ALL	Bivalent CAR	5	6	0.83	[0.36; 1.00]	2.8%
Liu 2021 B G1	Abstract	CD19/CD22	ALL	Tandem	NA	49			0.0%
Liu 2021 B G2	Abstract	CD19/CD22	ALL	Sequential Infusion	NA	13			0.0%
Pan 2020	Full-Report	CD19/CD22	ALL	Sequential Infusion	20	20	1.00	[0.83; 1.00]	4.3%
Schultz 2018	Abstract	CD19/CD22	ALL	Bivalent CAR	4	4	1.00	[0.40; 1.00]	2.2%
Shalabi 2020	Abstract	CD19/CD22	ALL	Bivalent CAR	8	11	0.73	[0.39; 0.94]	3.6%
Spiegel 2021 A	Full-Report	CD19/CD22	ALL	Bivalent CAR	17	17	1.00	[0.80; 1.00]	4.2%
Wang 2020 A	Full-Report	CD19/CD22	ALL	Sequential Infusion	49	51	0.96	[0.87; 1.00]	5.1%
Wang 2021	Full-Report	CD19/CD22	ALL	Coadministration	3	3	1.00	[0.29; 1.00]	1.8%
Wei 2021 A	Full-Report	CD19/CD22	ALL	Bivalent CAR	NA	15			0.0%
Yang 2018	Abstract	CD19/CD22	ALL	Cotransduction	15	15	1.00	[0.78; 1.00]	4.0%
Yang 2020	Abstract	CD19/CD22	ALL	Bivalent CAR	9	10	0.90	[0.55; 1.00]	3.5%
Yang 2019	Abstract	CD19/CD22	ALL	Bivalent CAR	8	11	0.73	[0.39; 0.94]	3.6%
**Random effects model**						**297**	**0.95**	**[0.89; 0.99]**	**45.8%**
Heterogeneity: $I^2 = 55\%$, $\tau^2 = 0.0233$, $p = 0.0621$									
**CD22 & NHL**									
Baird 2021	Full-Report	CD22	NHL	NA	18	21	0.86	[0.64; 0.97]	4.4%
Zhu 2021 B	Full-Report	CD22	NHL	NA	6	7	0.86	[0.42; 1.00]	3.0%
**Random effects model**						**28**	**0.86**	**[0.71; 0.96]**	**7.4%**
Heterogeneity: $I^2 = 0\%$, $\tau^2 = 0$, $p = 1.0000$									
**CD19/CD22 & NHL**									
Ramakrishnan 2020	Abstract	CD19/CD22	NHL	Bicistronic Vector	10	18	0.56	[0.31; 0.78]	4.2%
Spiegel 2021 B	Full-Report	CD19/CD22	NHL	Bivalent CAR	13	22	0.59	[0.36; 0.79]	4.4%
Wang 2020 B	Full-Report	CD19/CD22	NHL	Sequential Infusion	26	38	0.68	[0.51; 0.82]	4.9%
Wei 2021 B	Full-Report	CD19/CD22	NHL	Bivalent CAR	14	16	0.88	[0.62; 0.98]	4.1%
Zhang 2021 A	Full-Report	CD19/CD22	NHL	Bivalent CAR	23	32	0.72	[0.53; 0.86]	4.8%
Zhang 2021 B	Abstract	CD19/CD22	NHL	Sequential Infusion	9	11	0.82	[0.48; 0.98]	3.6%
**Random effects**						**137**	**0.70**	**[0.61; 0.79]**	**26.0%**
Heterogeneity: $I^2 = 26\%$, $\tau^2 = 0.0040$, $p = 0.2382$									
**Overall random effects model**						**582**	**0.85**	**[0.78; 0.91]**	**100.0%**
Heterogeneity: $I^2 = 73\%$, $\tau^2 = 0.0403$, $p < 0.0001$									
Test for subgroup differences: $\chi^2_3 = 22.59$, df = 3 ($p < 0.0001$)									

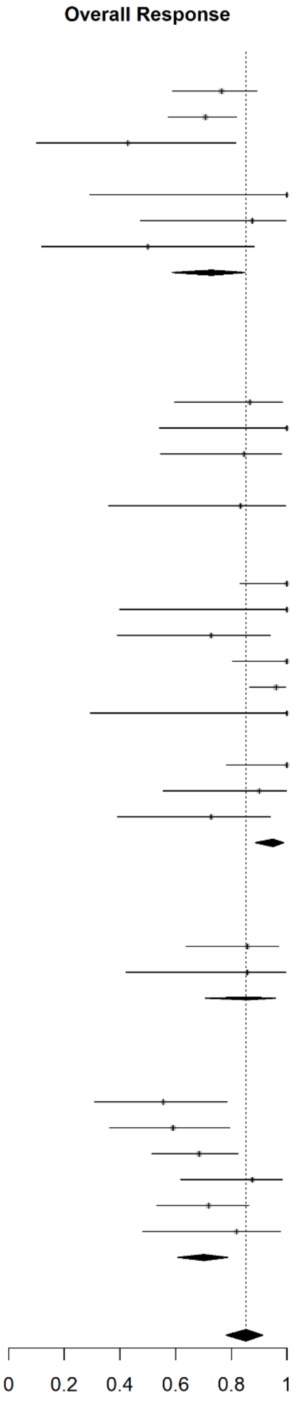

Supplemental Figure 6. Overall response incidence organized by disease type and antigen target.

PRISMA Checklist

Section and Topic	Item #	Checklist item	Location where item is reported
**TITLE**			
Title	1	Identify the report as a systematic review.	Page 1
**ABSTRACT**			
Abstract	2	See the PRISMA 2020 for Abstracts checklist.	Page 1-2
**INTRODUCTION**			
Rationale	3	Describe the rationale for the review in the context of existing knowledge.	Page 2
Objectives	4	Provide an explicit statement of the objective(s) or question(s) the review addresses.	Page 2 lines 68-70
**METHODS**			
Eligibility criteria	5	Specify the inclusion and exclusion criteria for the review and how studies were grouped for the syntheses.	Page 3
Information sources	6	Specify all databases, registers, websites, organisations, reference lists and other sources searched or consulted to identify studies. Specify the date when each source was last searched or consulted.	Page 3 lines 85-92
Search strategy	7	Present the full search strategies for all databases, registers and websites, including any filters and limits used.	Supplemental Materials Page 2
Selection process	8	Specify the methods used to decide whether a study met the inclusion criteria of the review, including how many reviewers screened each record and each report retrieved, whether they worked independently, and if applicable, details of automation tools used in the process.	Page 3
Data collection process	9	Specify the methods used to collect data from reports, including how many reviewers collected data from each report, whether they worked independently, any processes for obtaining or confirming data from study investigators, and if applicable, details of automation tools used in the process.	Page 3-4
Data items	10a	List and define all outcomes for which data were sought. Specify whether all results that were compatible with each outcome domain in each study were sought (e.g. for all measures, time points, analyses), and if not, the methods used to decide which results to collect.	Page 3-4; full list found in previously published protocol
	10b	List and define all other variables for which data were sought (e.g. participant and intervention characteristics, funding sources). Describe any assumptions made about any missing or unclear information.	Page 3-4; full list found in previously published protocol
Study risk of bias assessment	11	Specify the methods used to assess risk of bias in the included studies, including details of the tool(s) used, how many reviewers assessed each study and whether they worked independently, and if applicable, details of automation tools used in the process.	Page 54, RoB tool stated in lines 112-113
Effect measures	12	Specify for each outcome the effect measure(s) (e.g. risk ratio, mean difference) used in the synthesis or presentation of results.	Page 4 lines 114-129

Section and Topic	Item #	Checklist item	Location where item is reported
Synthesis methods	13a	Describe the processes used to decide which studies were eligible for each synthesis (e.g. tabulating the study intervention characteristics and comparing against the planned groups for each synthesis (item #5)).	NA
	13b	Describe any methods required to prepare the data for presentation or synthesis, such as handling of missing summary statistics, or data conversions.	Page 3-4, details in previous published protocol
	13c	Describe any methods used to tabulate or visually display results of individual studies and syntheses.	Page 4
	13d	Describe any methods used to synthesize results and provide a rationale for the choice(s). If meta-analysis was performed, describe the model(s), method(s) to identify the presence and extent of statistical heterogeneity, and software package(s) used.	Page 4
	13e	Describe any methods used to explore possible causes of heterogeneity among study results (e.g. subgroup analysis, meta-regression).	Page 4
	13f	Describe any sensitivity analyses conducted to assess robustness of the synthesized results.	Page 4
Reporting bias assessment	14	Describe any methods used to assess risk of bias due to missing results in a synthesis (arising from reporting biases).	Page 4
Certainty assessment	15	Describe any methods used to assess certainty (or confidence) in the body of evidence for an outcome.	Page 4
**RESULTS**			
Study selection	16a	Describe the results of the search and selection process, from the number of records identified in the search to the number of studies included in the review, ideally using a flow diagram.	Page 4; Figure 1
	16b	Cite studies that might appear to meet the inclusion criteria, but which were excluded, and explain why they were excluded.	Supplemental Materials Page 41-45
Study characteristics	17	Cite each included study and present its characteristics.	Table 1, Table 2, Table 3
Risk of bias in studies	18	Present assessments of risk of bias for each included study.	Page 8, Supplemental Figure 2, Supplemental Figure 3
Results of individual studies	19	For all outcomes, present, for each study: (a) summary statistics for each group (where appropriate) and (b) an effect estimate and its precision (e.g. confidence/credible interval), ideally using structured tables or plots.	Figure 2, Figure 3
Results of syntheses	20a	For each synthesis, briefly summarise the characteristics and risk of bias among contributing studies.	Page 8
	20b	Present results of all statistical syntheses conducted. If meta-analysis was done, present for each the summary estimate and its precision (e.g. confidence/credible interval) and measures of statistical heterogeneity. If comparing groups, describe the direction of the effect.	Figure 2, Figure 3, Figure 4
	20c	Present results of all investigations of possible causes of heterogeneity among study results.	Supplemental Table 5

Section and Topic	Item #	Checklist item	Location where item is reported
			Supplemental Table 1
	20d	Present results of all sensitivity analyses conducted to assess the robustness of the synthesized results.	Supplemental Figure 5
Reporting biases	21	Present assessments of risk of bias due to missing results (arising from reporting biases) for each synthesis assessed.	Supplemental Figure 4
Certainty of evidence	22	Present assessments of certainty (or confidence) in the body of evidence for each outcome assessed.	Supplemental Table 4
**DISCUSSION**			
Discussion	23a	Provide a general interpretation of the results in the context of other evidence.	Page 9
	23b	Discuss any limitations of the evidence included in the review.	Page 10 lines 342-346
	23c	Discuss any limitations of the review processes used.	Page 10 lines 342-346
	23d	Discuss implications of the results for practice, policy, and future research.	Page 9-10
**OTHER INFORMATION**			
Registration and protocol	24a	Provide registration information for the review, including register name and registration number, or state that the review was not registered.	Page 3 line 75-76
	24b	Indicate where the review protocol can be accessed, or state that a protocol was not prepared.	Page 3, References
	24c	Describe and explain any amendments to information provided at registration or in the protocol.	Page 4 (use of arcsine transformation instead of logit transformation)
Support	25	Describe sources of financial or non-financial support for the review, and the role of the funders or sponsors in the review.	Page 11
Competing interests	26	Declare any competing interests of review authors.	Page 11
Availability of data, code and other materials	27	Report which of the following are publicly available and where they can be found: template data collection forms; data extracted from included studies; data used for all analyses; analytic code; any other materials used in the review.	Supplementary Materials

From: Page MJ, McKenzie JE, Bossuyt PM, Boutron I, Hoffmann TC, Mulrow CD, et al. The PRISMA 2020 statement: an updated guideline for reporting systematic reviews. BMJ 2021;372:n71. doi: 10.1136/bmj.n71
For more information, visit: http://www.prisma-statement.org/

Included Studies and Associated Publications

Study ID	Associated Publications (Bold = Primary report)
Annesley 2021	**Annesley C, Summers C, Pulsipher MA, Skiles JL, Li AM, Vatsayan A, et al. SCRI-CAR19x22v2 T Cell Product Demonstrates Bispecific Activity in B-ALL. Blood. 2021;138(Supplement 1):470–470.**
Baird 2021	**Baird JH, Frank MJ, Craig J, Patel S, Spiegel JY, Sahaf B, et al. CD22-directed CAR T-cell therapy induces complete remissions in CD19-directed CAR–refractory large B-cell lymphoma. Blood. 2021;137(17):2321–5.**<br><br>Baird JH, Frank MJ, Craig J, Patel S, Spiegel JY, Sahaf B, et al. CD22-Directed CAR T-Cell Therapy Mediates Durable Complete Responses in Adults with Relapsed or Refractory Large B-Cell Lymphoma after Failure of CD19-Directed CAR T-Cell Therapy and High Response Rates in Adults with Relapsed or Refractory B-Cell Acute Lymphoblastic Leukemia. Blood. 2020;136(Supplement 1):28–9.<br><br>Frank MJ, Baird JH, Patel S, Craig J, Spiegel JY, Ehlinger Z, et al. CD22-CAR T-Cell Therapy Mediates High Durable Remission Rates in Adults with Large B-Cell Lymphoma Who Have Relapsed after CD19-CAR T-Cell Therapy. Blood. 2021;138(Supplement 1):741–741.
Cao 2021	**Cao Y, Xiao Y, Wang N, Wang G, Huang L, Hong Z, et al. CD19/CD22 Chimeric Antigen Receptor T Cell Cocktail Therapy following Autologous Transplantation in Patients with Relapsed/Refractory Aggressive B Cell Lymphomas. Transplantation and cellular therapy. 2021;27(11):910.e1–910.e11.**<br><br>Cao Y, Xiao Y, Wang N, Wang G, Zhou X, Huang L, et al. CD19/CD22 CAR-T Cell Cocktail Therapy Following Autologous Transplantation in Patients with Relapsed/Refractory B-Cell Lymphomas. Blood. 2020;136(Supplement 1):11–11.<br><br>Wei J, Mao Z, Wang N, Huang L, Cao Y, Sun W, et al. Long-term outcomes of relapsed/refractory double-hit lymphoma (r/r DHL) treated with CD19/22 CAR T-cell cocktail therapy. Clinical and translational medicine. 2020;10(5):e176–n/a.<br><br>Wu J, Meng F, Cao Y, Zhang Y, Zhu X, Wang N, et al. Sequential CD19/22 CAR T-cell immunotherapy following autologous stem cell transplantation for central nervous system lymphoma. Blood cancer journal (New York). 2021;11(7):131–131.<br><br>Cao Y, Wang N, Wang G, Xiao Y, Huang L, Li C, et al. Sequential Infusion of Anti-CD22 and Anti-CD19 Chimeric Antigen Receptor T Cells Following Autologous HSCT in Patients with B-NHL. Blood. 2018;132(Supplement 1):2054–2054.
Cordoba 2021	**Cordoba S, Onuoha S, Thomas S, Pignataro DS, Hough R, Ghorashian S, et al. CAR T cells with dual targeting of CD19 and CD22 in pediatric and young adult patients with relapsed or refractory B cell acute lymphoblastic leukemia: a phase 1 trial. Nature medicine. 2021;27(10):1797–805.**<br><br>Amrolia PJ, Wynn R, Hough RE, Vora A, Bonney D, Veys P, et al. Phase I Study of AUTO3, a Bicistronic Chimeric Antigen Receptor (CAR) T-Cell Therapy Targeting CD19 and CD22, in Pediatric Patients with Relapsed/Refractory B-Cell Acute Lymphoblastic Leukemia (r/r B-ALL): Amelia Study. Blood. 2019;134(Supplement_1):2620–2620.<br><br>Amrolia PJ, Wynn R, Hough R, Vora A, Bonney D, Veys P, et al. Simultaneous Targeting of CD19 and CD22: Phase I Study of AUTO3, a Bicistronic Chimeric Antigen Receptor (CAR) T-Cell Therapy, in Pediatric Patients with Relapsed/Refractory B-Cell Acute Lymphoblastic Leukemia (r/r B-ALL): Amelia Study. Blood. 2018;132(Supplement 1):279–279.
Dai 2020	**Dai H, Wu Z, Jia H, Tong C, Guo Y, Ti D, et al. Bispecific CAR-T cells targeting both CD19 and CD22 for therapy of adults with relapsed or refractory B cell acute lymphoblastic leukemia. Journal of hematology and oncology. 2020;13(1):30–10.**
Frey 2021	**Frey NV, Gill S, Hwang WT, Luger SM, Martin ME, McCurdy SR, et al. CART22-65s Co-Administered with huCART19 in Adult Patients with Relapsed or Refractory ALL. Blood. 2021;138(Supplement 1):469–469.**
Gardner 2020	**Gardner RA, Annesley C, Wilson A, Summers C, Narayanaswamy P, Wu V, et al. Efficacy of SCRI-CAR19x22 T cell product in B-ALL and persistence of anti-CD22 activity. Journal of clinical oncology. 2020;38(15_suppl):3035–3035.**<br><br>Gardner R, Annesley C, Finney O, Summers C, Lamble AJ, Rivers J, et al. Early Clinical Experience of CD19 x CD22 Dual Specific CAR T Cells for Enhanced Anti-Leukemic Targeting of Acute Lymphoblastic Leukemia. Blood. 2018;132(Supplement 1):278–278.
Hu 2021	**Hu Y, Zhou Y, Zhang M, Ge W, Li Y, Yang L, et al. CRISPR/Cas9-Engineered Universal CD19/CD22 Dual-Targeted CAR-T Cell Therapy for Relapsed/Refractory B-cell Acute Lymphoblastic Leukemia. Clinical cancer research. 2021;27(10):2764–72.**<br><br>Hu Y, Zhou Y, Zhang M, Ge W, Li Y, Yang L, et al. The Safety and Efficacy of a CRISPR/Cas9-Engineered Universal CAR-T Cell Product (CTA101) in Patients with Relapsed/Refractory B-Cell Acute Lymphoblastic Leukemia. Blood. 2020;136(Supplement 1):52–52.
Liu 2021 A	**Liu S, Deng B, Yin Z, Lin Y, An L, Liu D, et al. Combination of CD19 and CD22 CAR-T cell therapy in relapsed B-cell acute lymphoblastic leukemia after allogeneic transplantation. American journal of hematology. 2021;96(6):671–9.**
Liu 2021 B	**Liu S, Zhang X, Dai H, Cui Q, Cui W, Yin J, et al. Tandem CD19/CD22 Dual Targets CAR-T Cells Therapy Obtains Superior CR Rate Than**

	**Single CD19 CAR-T Cells Infusion As Well As Sequential CD19 and CD22 CAR-T Cells Infusion for Relapsed/Refractory B-Cell Acute Lymphoblastic Leukemia Patients. Blood. 2021;138(Supplement 1):1755–1755.**<br><br>Cui W, Zhang X, Dai H, Cui Q, Song B, Wu D, et al. Tandem CD19/CD22 Dual Targets CAR-T Cells Therapy Acquires Superior CR Rate Than CD19 CAR-T Cells: A Case Controlled Study. Blood. 2020;136(Supplement 1):44–44.<br><br>Ma Y, Qu C, Dai H, Liu S, Cui Q, Cui W, et al. Decitabine in Combination with Fludarabine and Cyclophosphamide As Lymphodepletion Regimen Followed By CD19/CD22 Bispecific Targeted CAR-T Therapy Significantly Improves Survival in Relapsed/Refractory B-ALL Patients: A Pilot Study. Blood. 2021;138(Supplement 1):1754–1754.<br><br>Zhang XY, Dai HP, Li Z, Yin J, Lang XP, Yang CX, et al. Identification of STRBP as a Novel JAK2 Fusion Partner Gene in a Young Adult With Philadelphia Chromosome-Like B-Lymphoblastic Leukemia. Frontiers in oncology. 2020;10:611467–611467.<br><br>Zhang XY, Dai H ping, Zhang L, Liu SN, Dai Y, Wu DP, et al. MRD-Negative Remission Induced in EP300-ZNF384 Positive B-ALL Patients by Tandem CD19/CD22 CAR T-Cell Therapy Bridging to Allogeneic Stem Cell Transplantation. OncoTargets and therapy. 2021;14:5197–204.
Liu 2022	**Liu Y, Deng B, Hu B, Zhang W, Zhu Q, Liu Y, et al. Sequential different B-cell antigen–targeted CAR T-cell therapy for pediatric refractory/relapsed Burkitt lymphoma. Blood advances. 2022;6(3):717–30.**<br><br>Zhang W, Yang J, Zhou C, Hu B, Jin L, Deng B, et al. Early response observed in pediatric patients with relapsed/refractory Burkitt lymphoma treated with chimeric antigen receptor T cells. Blood. 2020;135(26):2425–7.
Pan 2019	**Pan J, Niu Q, Deng B, Liu S, Wu T, Gao Z, et al. CD22 CAR T-cell therapy in refractory or relapsed B acute lymphoblastic leukemia. Leukemia. 2019;33(12):2854–66.**<br><br>Zhang Y, Chen H, Song Y, Tan X, Zhao Y, Liu X, et al. Chimeric antigens receptor T cell therapy as a bridge to haematopoietic stem cell transplantation for refractory/ relapsed B-cell acute lymphomalastic leukemia. British journal of haematology. 2020;189(1):146–52.
Pan 2020	**Pan J, Zuo S, Deng B, Xu X, Li C, Zheng Q, et al. Sequential CD19-22 CAR T therapy induces sustained remission in children with r/r B-ALL. Blood. 2020;135(5):387–91.**<br><br>Pan J, Tang K, Deng B, Ling Z, Song W, Chang A. LONG-TERM FOLLOW-UP OF SEQUENTIAL CD19-22 CAR T-CELL THERAY IN 20 CHILDREN WITH REFRACTORY OR RELAPSED B-ALL. EHA2021 Virtual Congress Abstract Book. HemaSphere. 2021;5(Suppl).
Ramakrishnan 2020	**Ramakrishnan A, Marzolini M, Osborne W, Tholouli E, Bachier C, McSweeney P, et al. Phase 1 Alexander Study of AUTO3 the First Bicistronic Chimeric Antigen Receptor (CAR) Targeting CD19 and CD22 with Pembrolizumab in Patients with Relapsed/Refractory Diffuse Large B Cell Lymphoma. Abstract Book: 25th Congress of the European Hematology Association Virtual Edition, 2020. HemaSphere. 2020;4(Suppl).**<br><br>Osborne W, Marzolini M, Tholouli E, Ramakrishnan A, Bachier CR, McSweeney PA, et al. Phase I Alexander study of AUTO3, the first CD19/22 dual targeting CAR T cell therapy, with pembrolizumab in patients with relapsed/refractory (r/r) DLBCL. Journal of clinical oncology. 2020;38(15_suppl):8001–8001.<br><br>Tholouli E, Osborne W, Bachier C, Ramakrishnan A, Marzolini M, Irvine D, et al. 890MO Phase I Alexander study of AUTO3, the first CD19/22 dual targeting CAR.T cell, with pembrolizumab in patients with relapsed/refractory (r/r) DLBCL. Annals of oncology. 2020;31:S651–S651.<br><br>Ardeshna K, Marzolini MAV, Osborne W, Al-Hajj M, Thomas S, Faulkner J, et al. Study of AUTO3, the First Bicistronic Chimeric Antigen Receptor (CAR) Targeting CD19 and CD22, Followed By Anti-PD1 Consolidation in Patients with Relapsed/Refractory (r/r) Diffuse Large B Cell Lymphoma (DLBCL): Alexander Study. Blood. 2018;132(Supplement 1):1679–1679.<br><br>Ardeshna KM, Marzolini MAV, Norman J, Al-Hajj M, Thomas S, Faulkner J, et al. Phase 1/2 Study of AUTO3 the First Bicistronic Chimeric Antigen Receptor (CAR) Targeting CD19 and CD22 Followed By an Anti-PD1 in Patients with Relapsed/Refractory (r/r) Diffuse Large B Cell Lymphoma (DLBCL): Results of Cohort 1 and 2 of the Alexander Study. Blood. 2019;134(Supplement_1):246–246.
Schultz 2018	**Schultz LM, Davis KL, Baggott C, Chaudry C, Marcy AC, Mavroukakis S, et al. Phase 1 Study of CD19/CD22 Bispecific Chimeric Antigen Receptor (CAR) Therapy in Children and Young Adults with B Cell Acute Lymphoblastic Leukemia (ALL). Blood. 2018;132(Supplement 1):898–898.**<br><br>Schultz LM, Muffly LS, Spiegel JY, Ramakrishna S, Hossain N, Baggott C, et al. Phase I Trial Using CD19/CD22 Bispecific CAR T Cells in Pediatric and Adult Acute Lymphoblastic Leukemia (ALL). Blood. 2019;134(Supplement_1):744–744.
Shah 2020	**Shah NN, Highfill SL, Shalabi H, Yates B, Jin J, Wolters PL, et al. CD4/CD8 T-Cell Selection Affects Chimeric Antigen Receptor (CAR) T-Cell Potency and Toxicity: Updated Results From a Phase I Anti-CD22 CAR T-Cell Trial. Journal of clinical oncology. 2020;38(17):1938–50.**<br><br>Ishii K, Pouzolles M, Chien CD, Erwin-Cohen RA, Kohler ME, Qin H, et al. Perforin-deficient CAR T cells recapitulate late-onset inflammatory toxicities observed in patients. The Journal of clinical investigation. 2020;130(10):5425–43.

	Jess J, Yates B, Dulau-Florea A, Cullinane A, Shalabi H, Lozier J, Shah N. Hematopoietic toxicities and coagulopathy following CD22 CAR T-cells. Pediatric Blood and Cancer. 2021;68():S196-S197.<br><br>Lichtenstein DA, Schischlik F, Shao L, Steinberg SM, Yates B, Wang HW, et al. Characterization of HLH-like manifestations as a CRS variant in patients receiving CD22 CAR T cells. Blood. 2021;138(24):2469–84.<br><br>Lichtenstein DA, Steinberg SM, Highfill SL, Yates B, Jin P, Jin J, et al. Abstract 4231: Factors predictive of CAR T cell associated hemophagocytic lymphohistiocytosis (HLH). Cancer research (Chicago, Ill). 2020;80(16_Supplement):4231–4231.<br><br>Masih KE, Ligon JA, Yates B, Shalabi H, Little L, Islam Z, et al. Consequences of hemophagocytic lymphohistiocytosis-like cytokine release syndrome toxicities and concurrent bacteremia. Pediatric blood & cancer. 2021;68(10):e29247–n/a.<br><br>Mikkilineni L, Yates B, Steinberg SM, Shahani SA, Molina JC, Palmore T, et al. Infectious complications of CAR T-cell therapy across novel antigen targets in the first 30 days. Blood advances. 2021;5(23):5312–22.<br><br>Ombrello A, Yates B, Shalabi H, Fry T, Shah N. Experience with and management of HLH-like toxicities following chimeric antigen receptor T-cell therapy for treatment of relapsed/ refractory pre-B ALL. Arthritis and Rheumatology. 2020;72(Supplement 1):16-17.<br><br>Shah NN, Stetler-Stevenson M, Yuan CM, Shalabi H, Yates B, Delbrook C, et al. Minimal Residual Disease Negative Complete Remissions Following Anti-CD22 Chimeric Antigen Receptor (CAR) in Children and Young Adults with Relapsed/Refractory Acute Lymphoblastic Leukemia (ALL). Blood. 2016;128(22):650–650.<br><br>Shalabi H, Wolters PL, Martin S, Delbrook C, Yates B, Lee DW, et al. A Prospective Evaluation of Neurocognitive Function and Neurologic Symptoms in Pediatric and Young Adult Patients with Relapsed/Refractory Acute Lymphoblastic Leukemia (ALL) Undergoing Anti-CD22 Chimeric Antigen Receptor Therapy. Blood. 2016;128(22):1625–1625.<br><br>Ishii K, Shalabi H, Yates B, Delbrook C, Mackall CL, Fry TJ, et al. Tocilizumab-Refractory Cytokine Release Syndrome (CRS) Triggered By Chimeric Antigen Receptor (CAR)-Transduced T Cells May Have Distinct Cytokine Profiles Compared to Typical CRS. Blood. 2016;128(22):3358–3358.<br><br>Shah NN, Highfill SL, Shalabi H, Yates B, Kane E, Fellowes V, et al. CD4/CD8 T-Cell Selection Enhances CD22 CAR-T Cell Transduction and in-Vivo CAR-T Expansion: Updated Results on Phase I Anti-CD22 CAR Dose Expansion Cohort. Blood. 2017;130(Suppl_1):809–809.<br><br>Shalabi H, Shah NN, Fry TJ, Yates B, Delbrook C. Chimeric Antigen Receptor Induced Cytopenia Differs from Chemotherapy Induced Myelosuppression. Blood. 2017;130:5048–5048.<br><br>Shah NN, Shalabi H, Yates B, Kane E, Dulau-Florea A, Cullinane A, et al. Beyond Cytokine Storm: Optimizing Treatment Strategies to Target the Complex Interplay between CAR Mediated Inflammatory Response, Disseminated Intravascular Coagulation and Macrophage Activation Syndrome. Blood. 2017;130:1277–1277.<br><br>Shalabi H, Shah NN, Fry TJ, Yates B, Delbrook C, Yuan C, et al. Intensification of Lymphodepletion Optimizes CAR Re-Treatment Efficacy. Blood. 2017;130:3889–3889.<br><br>Shalabi H, Delbrook C, Stetler-Stevenson M, Yuan C, Yates B, Fry TJ, Shah NN. Chimeric Antigen Receptor t-cell (CAR-T) therapy can render patients with all into PCR-negative remission and can be an effective bridge to transplant (HCT). Pediatric Blood and Cancer. 2018;65(Supplement 1):S292-S293<br><br>Yates B, Shalabi H, Civelek AC, Delbrook C, Fry TJ, Shah NN. Efficacy and Kinetics of CAR-T Cell Therapy in Children and Young Adults with Extramedullary Acute Lymphoblastic Leukemia (ALL) and Non-Hodgkin Lymphoma (NHL). Biology of blood and marrow transplantation. 2018;24(3):S75–S76.<br><br>Shalabi H, Delbrook C, Stetler-Stevenson M, Yuan C, Steinberg SM, Yates B, et al. Chimeric Antigen Receptor T-Cell (CAR-T) Therapy Can Render Patients with ALL Into PCR-Negative Remission and Can be an Effective Bridge to Transplant (HCT). Biology of blood and marrow transplantation. 2018;24(3):S25–S26.<br><br>Shah NN, Qin H, Yates B, Su L, Shalabi H, Raffeld M, et al. Clonal expansion of CAR T cells harboring lentivector integration in the CBL gene following anti-CD22 CAR T-cell therapy. Blood advances. 2019;3(15):2317–22.<br><br>Mikkilineni L, Shahani S, Yates B, Steinberg SM, Palmore T, Nussenblatt V, et al. Infectious Complications Associated with CAR T-Cell Therapy. Blood. 2019;134(Supplement_1):4449–4449.<br><br>Fry TJ, Stetler-Stevenson M, Shah NN, Yuan CM, Yates B, Delbrook C, et al. Clinical Activity and Persistence of Anti-CD22 Chimeric Antigen

	Receptor in Children and Young Adults with Relapsed/Refractory Acute Lymphoblastic Leukemia (ALL). Blood. 2015;126(23):1324–1324.<br><br>Fry TJTJ, Shah NNNN, Orentas RJRJ, Stetler-Stevenson MM, Yuan CMCM, Ramakrishna SS, et al. CD22-CAR T Cells Induce Remissions in CD19-CAR Naïve and Resistant B-ALL. Nature medicine. 2017;24(1):20–8.<br><br>Shalabi H, Kraft IL, Wang HW, Yuan CM, Yates B, Delbrook C, et al. Sequential loss of tumor surface antigens following chimeric antigen receptor T-cell therapies in diffuse large B-cell lymphoma. Haematologica (Roma). 2018;103(5):e215–e218.<br><br>Shalabi H, Wolters PL, Martin S, Toledo-Tamula MA, Roderick MC, Struemph K, et al. Systematic Evaluation of Neurotoxicity in Children and Young Adults Undergoing CD22 Chimeric Antigen Receptor T-Cell Therapy. Journal of immunotherapy (1997). 2018;41(7):350–8.<br><br>Ombrello A, Yates B, Shalabi H, Fry TJ, Shah NN. Experience with and management of HLH-like toxicities following chimeric antigen receptor T-cell therapy for treatment of relapsed/ refractory pre-B ALL. Pediatric Rheumatology. 2019;17(Supplement 1).<br><br>Shah NN, Shalabi H, Yates B, Yuan C, Qin H, Ombrello A, et al. Abstract LB-146: Phase I CD22 CAR T-cell trial updates. Cancer research (Chicago, Ill). 2019;79(13_Supplement):LB–146–LB–146.<br><br>Shalabi H, Koegel A, Ponduri A, Qin H, Salem D, Stetler-Stevenson M, et al. Case report: Impact of BITE on CAR-T cell expansion. Advances in cell and gene therapy. 2019;2(3).<br><br>Yates B, Shalabi H, Salem D, Delbrook C, Yuan CM, Stetler-Stevenson M, et al. Sequential CD22 Targeting Impacts CD22 CAR-T Cell Response. Blood. 2018;132(Supplement 1):282–282.
Shalabi 2020	**Shalabi H, Yates B, Shahani S, Qin H, HIghfill SL, Panch S, et al. Abstract CT051: Safety and efficacy of CD19/CD22 CAR T cells in children and young adults with relapsed/refractory ALL. Cancer research (Chicago, Ill). 2020;80(16_Supplement):CT051–CT051.**<br><br>Holland EM, Yates B, Ling A, Yuan CM, Wang HW, Stetler-Stevenson M, et al. Characterization of extramedullary disease in B-ALL and response to CAR T-cell therapy. Blood advances. 2022;6(7):2167–82.<br><br>Molina JC, Steinberg SM, Yates B, Lee DW, Little L, Mackall CL, et al. Factors Impacting Overall and Event-Free Survival following Post-Chimeric Antigen Receptor T Cell Consolidative Hematopoietic Stem Cell Transplantation. Transplantation and cellular therapy. 2022;28(1):31.e1–31.e9.
Singh 2021	**Singh N, Frey NV, Engels B, Barrett DM, Shestova O, Ravikumar P, et al. Antigen-independent activation enhances the efficacy of 4-1BB-costimulated CD22 CAR T cells. Nature medicine. 2021;27(5):842–50.**<br><br>Ruella M, Maude SL, Engels B, Barrett DM, Frey N, Marcucci KT, et al. Clinical Efficacy of Anti-CD22 Chimeric Antigen Receptor T Cells for B-Cell Acute Lymphoblastic Leukemia Is Correlated with the Length of the Scfv Linker and Can be Predicted Using Xenograft Models. Blood. 2017;130(Suppl_1):807–807.<br><br>Singh N, Frey NV, Engels B, Barrett DM, Shestova O, Ravikumar P, et al. Single Chain Variable Fragment Linker Length Regulates CAR Biology and T Cell Efficacy. Blood. 2019;134(Supplement_1):247–247.
Speigel 2021	**Spiegel JY, Patel S, Muffly L, Hossain NM, Oak J, Baird JH, et al. CAR T cells with dual targeting of CD19 and CD22 in adult patients with recurrent or refractory B cell malignancies: a phase 1 trial. Nature medicine. 2021;27(8):1419–31.**<br><br>Hossain N, Sahaf B, Abramian M, Spiegel JY, Kong K, Kim S, et al. Phase I Experience with a Bi-Specific CAR Targeting CD19 and CD22 in Adults with B-Cell Malignancies. Blood. 2018;132(Supplement 1):490–490.
Summers 2021	**Summers C, Baxter B, Annesley C, Yokoyama J, Rhea S, Huang W, et al. CD22 CAR Optimization for Improved in-Human Activity Following Inadequate CD22 CAR Activity in Phase 1 Clinical Trial PLAT-04. Blood. 2021;138(Supplement 1):403–403.**
Tan 2021	**Tan Y, Cai H, Li C, Deng B, Song W, Ling Z, et al. A novel full-human CD22-CAR T cell therapy with potent activity against CD22low B-ALL. Blood cancer journal (New York). 2021;11(4):71–71.**
Wang 2020	**Wang N, Hu X, Cao W, Li C, Xiao Y, Cao Y, et al. Efficacy and safety of CAR19/22 T-cell cocktail therapy in patients with refractory/relapsed B-cell malignancies. Blood. 2020;135(1):17–27.**<br><br>Li T, Zhao L, Zhang Y, Xiao Y, Wang D, Huang L, et al. CAR T-Cell Therapy Is Effective but Not Long-Lasting in B-Cell Lymphoma of the Brain. Frontiers in oncology. 2020;10<br><br>Wei J, Cao W, Wang N, Xu H, Xiao M, Cao Y, Li C et al. Entecavir prophylaxis for hepatitis b virus reactivation in patients with CAR t-cell therapy. HemaSphere. 2020;4(Supplement 1):697.<br><br>Wei J, Xiao M, Mao Z, Wang N, Li C, Cao Y, et al. Anti CD19/22 Cocktail CAR T-Cell Therapy Can Improve the Outcomes of Patients with TP53-Mutated Relapsed/Refractory B-Cell Lymphoma. Blood. 2020;136(Supplement 1):43–43.

	Yan N, Wang N, Wang G, Huang L, Li C, Wang D, et al. CAR19/22 T cell cocktail therapy for B-ALL relapsed after allogeneic hematopoietic stem cell transplantation. Cytotherapy (Oxford, England). 2022;24(8):841–9.<br><br>Huang L, Wang N, Li C, Cao Y, Xiao Y, Xiao M, et al. Sequential Infusion of Anti-CD22 and Anti-CD19 Chimeric Antigen Receptor T Cells for Adult Patients with Refractory/Relapsed B-Cell Acute Lymphoblastic Leukemia. Blood. 2017;130(Suppl_1):846–846.<br><br>Huang L, Wang N, Cao Y, Li C, Xiao Y, Xiao M, et al. CAR22/19 Cocktail Therapy for Patients with Refractory/Relapsed B-Cell Malignancies. Blood. 2018;132(Supplement 1):1408–1408.<br><br>Wei J, Zhu X, Mao X, Huang L, Meng F, Zhou J. Severe early hepatitis B reactivation in a patient receiving anti-CD19 and anti-CD22 CAR T cells for the treatment of diffuse large B-cell lymphoma. Journal for immunotherapy of cancer. 2019;7(1):315–315.<br><br>Zeng C, Cheng J, Li T, Huang J, Li C, Jiang L, et al. Efficacy and toxicity for CD22/CD19 chimeric antigen receptor T-cell therapy in patients with relapsed/refractory aggressive B-cell lymphoma involving the gastrointestinal tract. Cytotherapy (Oxford, England). 2020;22(3):166–71.<br><br>Chen L, Xu B, Long X, Gu J, Lou Y, Wang D, et al. CAR T-cell therapy for a relapsed/refractory acute B-cell lymphoblastic lymphoma patient in the context of Li-Fraumeni syndrome. Journal for immunotherapy of cancer. 2020;8(1):e000364–.<br><br>Huang L, Wang N, Li C, Xiao Y, Cao Y, Xiao M, et al. Efficacy and safety of CAR19/22 T-cell “cocktail” therapy in patients with refractory/relapsed B-cell non-Hodgkin lymphoma. Journal of clinical oncology. 2019;37(15_suppl):2534–2534.
Wang 2021	**Wang T, Wan X, Yang F, Shi W, Liu R, Ding L, et al. Successful Treatment of TCF3-HLF–positive Childhood B-ALL with Chimeric Antigen Receptor T-Cell Therapy. Clinical lymphoma, myeloma and leukemia. 2021;21(6):386–92.**<br><br>Wang Y, Yang Y, Hong R, Zhao H, Wei G, Wu W, et al. A retrospective comparison of CD19 single and CD19/CD22 bispecific targeted chimeric antigen receptor T cell therapy in patients with relapsed/refractory acute lymphoblastic leukemia. Blood cancer journal (New York). 2020;10(10):105–105.
Wei 2021	**Wei G, Zhang Y, Zhao H, Wang Y, Liu Y, Liang B, et al. CD19/CD22 Dual-Targeted CAR T-cell Therapy for Relapsed/Refractory Aggressive B-cell Lymphoma: A Safety and Efficacy Study. Cancer immunology research. 2021;9(9):1061–70.**<br><br>Zhao HL, Hu YX, Nagler a, Chang AH, Huang H. CD19/CD22 Dual Targeted (Chimeric Antigen Receptor) CAR-T Therapy for Relapsed or RE Fractory (R/R) B-CELL Non-Hodgkin Lymphoma (B-NHL). Bone Marrow Transplantation. 2020;55(SUPPL 1):44-45.
Yang 2018	**Yang J, Li J, Zhang X, LV F, Guo X, Wang Q, et al. A Feasibility and Safety Study of CD19 and CD22 Chimeric Antigen Receptors-Modified T Cell Cocktail for Therapy of B Cell Acute Lymphoblastic Leukemia. Blood. 2018;132(Supplement 1):277–277.**
Yang 2019	**Yang J, Jiang P, Zhang X, Zhu X, Dong Q, He J, et al. Anti-CD19/CD22 Dual CAR-T Therapy for Refractory and Relapsed B-Cell Acute Lymphoblastic Leukemia. Blood. 2019;134(Supplement_1):284–284.**
Yang 2020	**Yang J, Jiang P, Zhang X, Li J, Wu Y, Xu L, et al. Successful 24-Hours Manufacture of Anti-CD19/CD22 Dual Chimeric Antigen Receptor (CAR) T Cell Therapy for B-Cell Acute Lymphoblastic Leukemia (B-ALL). Blood. 2020;136(Supplement 1):2–3.**
Zhang 2021 A	**Zhang Y, Li J, Zong X, Zhou J, Jia S, Geng H, et al. Chimeric Antigen Receptor T Cell Targeting to CD19 and CD22 Combined with Anti-PD-1 Antibody Induce High Response in Patients with Relapsed or Refractory B-Cell Non-Hodgkin Lymphoma. Blood. 2021;138(Supplement 1):1730–1730.**
Zhang 2021 B	**Zhang Y, Li J, Lou X, Chen X, Yu Z, Kang L, et al. A Prospective Investigation of Bispecific CD19/22 CAR T Cell Therapy in Patients With Relapsed or Refractory B Cell Non-Hodgkin Lymphoma. Frontiers in oncology. 2021;11:664421–664421.**
Zhu 2021	**Zhu H, Deng H, Mu J, Lyu C, Jiang Y, Deng Q. Anti-CD22 CAR-T Cell Therapy as a Salvage Treatment in B Cell Malignancies Refractory or Relapsed After Anti-CD19 CAR-T therapy. OncoTargets and therapy. 2021;14:4023–37.**
**Case Reports**	
Fu 2020	**Fu XH, Wang Y, Wang HJ, Wei SN, Xu YX, Xing HY, et al. CD19 antigen loss after treatment of Bispecific T-cell Engager and effective response to salvage bispecific CAR-T therapy in B cell acute lymphoblastic leukemia: a case report and literature review. Zhōnghuá xuèyèxué zázhì 2020;41(4):282–6.**
Hua 2020	**Hua J, Qian W, Wu X, Zhou L, Yu L, Chen S, et al. Sequential Infusion of Anti-CD22 and Anti-CD19 Chimeric Antigen Receptor T Cells for a Pediatric Ph-Like B-ALL Patient That Relapsed After CART-Cell and Haplo-HSCT Therapy: A Case Report and Review of Literature. OncoTargets and therapy. 2020;13:2311–7.**
Jain 2021	**Jain N, Roboz GJ, Konopleva M, Liu H, Schiller GJ, Jabbour EJ, et al. Preliminary Results from the Flu/Cy/Alemtuzumab Arm of the Phase I BALLI-01 Trial of UCART22, an Anti-CD22 Allogeneic CAR-T Cell Product, in Adult Patients with Relapsed or Refractory (R/R) CD22+ B-Cell Acute Lymphoblastic Leukemia (B-ALL). Blood. 2021;138(Supplement 1):1746–1746.**<br><br>Jain N, Roboz GJ, Konopleva M, Liu H, Jabbour E, Poirot C, et al. Preliminary Results of Balli-01: A Phase I Study of UCART22 (allogeneic

	engineered T-cells expressing anti-CD22 Chimeric Antigen Receptor) in Adult Patients with Relapsed or Refractory (R/R) CD22+ B-Cell Acute Lymphoblastic Leukemia (B-ALL). Blood. 2020;136(Supplement 1):7–8.
Jiao 2021	**Jiao C, Zvonkov E, Lai X, Zhang R, Liu Y, Qin Y, et al. 4SCAR2.0: a multi-CAR-T therapy regimen for the treatment of relapsed/refractory B cell lymphomas. Blood cancer journal (New York). 2021;11(3):59–59.**
Jin 2020	**Jin A, Feng J, Wei G, Wu W, Yang L, Xu H, et al. CD19/CD22 chimeric antigen receptor T-cell therapy for refractory acute B-cell lymphoblastic leukemia with FLT3-ITD mutations. Bone marrow transplantation (Basingstoke). 2020;55(4):717–21.**
Lai 2017	**Lai X, Gu X, Tsao ST, Zhang Q, Liu YC, Yaxian J, et al. Double CD19/CD22 Chimeric Antigen Receptor-Modified T Cells for the Treatment of Stage IV Relapsed and Refractory Follicular Lymphoma. Blood. 2017;130:5154–5154.**
Li 2019	**Li C, Zhang C, Chen X, Zhang Y, Chen J, Kang L, et al. Relative Depletion of Soluble Interleukin 6 Receptors Abolished the Development of Cytokine Release Syndrome after CART19/22 and Lenalidomide Treatment for Lymphoma. Blood. 2019;134(Supplement_1):5313–5313.**
Li 2019B	**Li N, Wang SA, Lin P, Jabbour E, Thompson P, Chen Z, et al. Relapsed B-acute lymphoblastic leukemia with aberrant myeloperoxidase expression following CAR T-cell therapy: A diagnostic challenge. American journal of hematology. 2019;94(9):1049–51.**
Liang 2020	**Liang Z, Cui J, Chang AH, Yu J, Hu Y, Huang H. Successful treatment of relapsed acute B-cell lymphoblastic leukemia with CD20/CD22 bispecific chimeric antigen receptor T-cell therapy. Regenerative therapy. 2020;15:281–4.**
Meijing 2019	**Meijing L, Cui R, Mu J, Yuan J jun, Mou N, Yang Z xing, et al. A Refractory B-ALL Patient Relapsed with Leukemia Cells Expressing CD19 and Anti-CD19 CAR Gene 14 Days after CR from Anti-CD19 CAR T-Cell Therapy. Blood. 2019;134(Supplement_1):5124–5124.**
Sun 2021	**Sun Y, Su Y, Wang Y, Liu N, Li Y, Chen J, et al. CD19 CAR-T Cells With Membrane-Bound IL-15 for B-Cell Acute Lymphoblastic Leukemia After Failure of CD19 and CD22 CAR-T Cells: Case Report. Frontiers in immunology. 2021;12:728962–.**
Wei 2020	**Wei S, Gu R, Xu Y, Liu X, Xing Y, Gong X, et al. Adjuvant ruxolitinib therapy relieves steroid-refractory cytokine-release syndrome without impairing chimeric antigen receptor-modified T-cell function. Immunotherapy. 2020;12(14):1047–52.**
Yan 2021	**Yan N, Wang N, Zhang P, Wang G, Mao X, Peng D, et al. Case Report: Successful Chimeric Antigen Receptor T Cell Therapy in Haploidentical-Allogeneic Stem Cell Transplant Patients With Post-Transplant Lymphoproliferative Disorder. Frontiers in oncology. 2021;11.**
Zhang 2019	**Zhang WQ, Hu B, Jin L, Yang J, Du J, Wang S, et al. CHIMERIC ANTIGEN RECEPTOR T-CELLS (CAR-T) FOR REFRACTORY AND RELAPSED BURKITT'S LYMPHOMA: EARLY RESPONSE IN PEDIATRIC PATIENTS. Hematological oncology. 2019;37(S2):59–60.**<br><br>Zhang WQ, Hu B, Jing L, Yang J, Du J, Wang S, et al. PF299 EARLY RESPONSE OBSERVATION OF PEDIATRIC PATIENTS WITH BURKITT'S LYMPHOMA TREATED WITH CHIMERIC ANTIGEN RECEPTOR (CAR) T-CELLS. HemaSphere. 2019;3(S1):101–2.<br><br>Du J, Zhang Y. Correction to: Sequential anti-CD19, 22, and 20 autologous chimeric antigen receptor T-cell (CAR-T) treatments of a child with relapsed refractory Burkitt lymphoma: a case report and literature review. Journal of cancer research and clinical oncology. 2020;146(8):2177–2177.<br><br>Du J, Zhang Y. Sequential anti-CD19, 22, and 20 autologous chimeric antigen receptor T-cell (CAR-T) treatments of a child with relapsed refractory Burkitt lymphoma: a case report and literature review. Journal of cancer research and clinical oncology. 2020;146(6):1575–82.
Zi 2021	**Zi FM, Ye LL, Zheng JF, Cheng J, Wang QM. Using JAK inhibitor to treat cytokine release syndrome developed after chimeric antigen receptor T cell therapy for patients with refractory acute lymphoblastic leukemia: A case report. Medicine (Baltimore). 2021;100(19):e25786–e25786.**

Studies Excluded at Full-Text Screening Stage

Reference	Reason
Hu Y, Houli Z, Guoqing W, Luo Y, Shi JM, Chen J, et al. Pre-Transplant MRD Negativity Predicts Favorable Outcomes of CAR-T Therapy Followed By Haploidentical HSCT for Relapsed/Refractory Acute Lymphoblastic Leukemia: A Multi-Center Retrospective Study. Blood. 2019;134(Supplement_1):3213–3213.	Did not state CAR target antigen
Lin Y, Deng B, Yin Z, An L, Zhao D, Liu D. Tyrosine kinase inhibitor followed by cart therapy for the treatment of relapsed philadelphia chromosome-positive B-cell acute lymphoblastic leukemia after allogeneic hematopoietic stem cell transplantation. Bone Marrow Transplantation. 2022;2020;55:264–5.	
Mohammad Y, Ali A, Allahyani B, Bassal M. Rapidly progressive vision loss following CAR T-cell therapy. [Abstract]. British journal of haematology. 2020;189(S1):4–294.	
Puri P, Severson KJ, Patel MH, Brumfiel CM, DiCaudo DJ, Rosenthal AC, et al. A case of B-cell lymphoblastic leukemia cutis flaring following CAR T-cell therapy in a patient with refractory acute lymphoblastic leukemia. JAAD Case Reports. 2021;8:64–6.	
Yang F, Shi H, Lei Y, Li R, Xu T, Deng B, et al. Allogeneic Hematopoietic Stem Cell Transplantation with Conditioning Including Donor Humanized CAR-T Cells for Refractory/Relapsed B-Cell Non-Hodgkin Lymphoma and Multiple Myeloma. Blood. 2021;138(Supplement 1):557–557.	
Chang LJ, Li Y, Tu S, Lai X, Huang R, Tsao ST, et al. Phase I/II Trial of Multi-Target Chimeric Antigen Receptor Modified T Cells (4SCAR2.0) Against Relapsed or Refractory Lymphomas. Blood. 2018;132(Supplement 1):225–225.	No clinical trial number stated
Jing P, Niu Q, Deng B, Liu S, Wu T, Gao Z, Chang A.H, Feng X, Tong C. CD22 CAR T-cell therapy in refractory or relapsed B acute lymphoblastic leukemia. 17th International Congress of Immunology, 19–23 October 2019, Beijing, China. European journal of immunology. 2019;49(S3):1723.	
Liu S, Deng B, Lin Y, Yin Z, Pan J, Wu T, et al. Sequential CD19- and CD22-CART Cell Therapies for Relapsed B-Cell Acute Lymphoblastic Leukemia after Allogeneic Hematopoietic Stem Cell Transplantation. Blood. 2018;132(Supplement 1):2126–2126.	
Tang X, Kang L, Qi W, Cui W, Dai H, Li Z, et al. Tandem CAR T Cells Targeting CD19 and CD22 Is a Safe and Highly Efficacious Treatment for Relapse/ Refractory ALL Patients. Blood. 2019;134(Supplement_1):1338–1338.	
Jiang YL, Li Q, Pu YD, Jiang YY, Yuan T, Deng Q, et al. Maintenance therapy following CD19 CAR-T treatment for relapsed B-cell acute lymphoblastic leukemia after allogeneic hematopoietic stem cell transplantation. Zhōnghuá xuèyèxué zázhì 2020;41(6):495–501.	
Feng Z, Hui L. CD22-Targeted chimeric antigen receptor T-cell therapy for adults with refractory or relapsed B acute lymphoblastic leukemia. EHA2021 Virtual Congress Abstract Book. HemaSphere. 2021;5(Suppl):e566.	
Wei J, Xiao M, Mao Z, Cao Y, Xiao Y, Meng F, et al. Outcomes of Relapsed/Refractory Aggressive B-Cell Non-Hodgkin Lymphoma (r/r B-NHL) Patients with TP53 Gene Disruption Treated with CD19/22 Cocktail CAR T-Cell Therapy Alone or Incorporated with Autologous Stem Cell Transplantation (ASCT). Blood. 2021;138(Supplement 1):94–94.	
Yang F, Hou R, Feng Y, Feng S, Geng Y, Shi H, Liu H, Li R, Wu T, Hu K. Haploidentical hematopoietic stem cell transplantation with conditioning including allogeneic bispecific car-T cells targeting both CD19 and CD22 for refractory/relapsed B-cell nonhodgkin lymphoma. EHA2021 Virtual Congress Abstract Book. HemaSphere. 2021;5(SUPPL 2):215-216	
Pan J, Tan Y, Deng B, Ling Z, Song W, Xu J, Duan J, Wang Z, Chang AH. High efficacy and safety of ruxolitinib in treating steroids resistant CRS during car T therapy, a pilot study. Abstract Book: 25th Congress of the European Hematology Association Virtual Edition, 2020. HemaSphere. 2020;4(Supplement 1):154.	
Qu C, Song Y, Yin J, et al. Decitabine may improve CAR-T efficacy in refractory/relapsed acute leukemia patients carrying TP53 alterations. Bone Marrow Transplant. 2021;56(7):1710-1713. doi:10.1038/s41409-021-01230-z	
Gao LL, Huang L, Wang N, Wang GX, Zhou XX, Li TJ, et al. Anti-CD22 CAR-T combined with anti-CD19 CAR-T cells in the treatment of relapsed or refractory acute B lymphocytic leukemia with severe cytokine release syndrome: two cases report and literature review. Zhōnghuá xuèyèxué zázhì 2019;40(9):780–2.	
Jia H, Wang Z, Wang Y, Liu Y, Dai H, Tong C, et al. Haploidentical CD19/CD22 bispecific CAR-T cells induced MRD-negative remission in a patient with relapsed and refractory adult B-ALL after haploidentical hematopoietic stem cell transplantation. Journal of hematology and oncology. 2019;12(1):57–57.	
Wei J, Mao Z, Sun W, et al. Jia Wei; , Zekai Mao; , Weimin Sun; ,Abstract Book: 25th Congress of the European Hematology Association Virtual Edition, 2020. HemaSphere. 2020;4(S1):693. CAR T-cell therapy can improve the outcome of relapsed/refractory double-hit lymphoma.	
Mao X, He C, Liu SY, Wang CY, Zhu L, Wang N, et al. Immunophenotypic changes after central recurrence in a case of Ph positive B-ALL receiving sequential infusion of anti-CD22 and anti-CD19 CAR-T therapy. Zhōnghuá xuèyèxué zázhì 2019;40(3):246–246.	
Yang F, Zhang J, Zhang X, Tian M, Wang J, Kang L, et al. Delayed remission following sequential infusion of humanized CD19- and CD22-modified CAR-T cells in a patient with relapsed/refractory acute lymphoblastic leukemia and prior exposure to murine-derived CD19-directed CAR-T cells. OncoTargets and therapy. 2019;12:2187–91.	
Cui W, Zhang X, Dai H, Cui Q, Yin J, Li Z, et al. Tandem CD19/CD22 Dual Targets CAR T-Cells Bridging Hematopoietic Stem Cells Transplantation	

Acquires Robust Remission for Relapsed and Refractory B Acute Lymphoblastic Leukemia Patients. Blood. 2021;138(Supplement 1):1753–1753.	
Hu Y, Zhang Y, Zhao H, Wang Y, Nagler A, Chang AH, et al. CD19/CD22 Dual-Targeted Chimeric Antigen Receptor T-Cell Therapy for Relapsed/Refractory Aggressive B-Cell Lymphoma: a Safety and Efficacy Study. Blood. 2020;136(Supplement 1):34–34.	
Huang C, Zhang HC, Ho JY, Liu RX, Wang L, Kuang N, et al. Dual specific CD19/CD22-targeted chimeric antigen receptor T-cell therapy for refractory diffuse large B-cell lymphoma: A case report. Oncology letters. 2020;20(4):1–21.	
Ma Y, Yang L, Bao Y, Yang Y, Chen L, Zheng M. Case Report: Post-CAR-T Infusion HBV Reactivation in Two Lymphoma Patients Despite Entecavir Preventive Therapy. Frontiers in immunology. 2021;12:751754.	
Niu ZY, Sun L, Wen SP, Song ZR, Xing L, Wang Y, et al. Programmed cell death protein-1 inhibitor combined with chimeric antigen receptor T cells in the treatment of relapsed refractory non-Hodgkin lymphoma: A case report. World journal of clinical cases. 2021;9(10):2394–9.	
Wang J, Shang Z, Wang J, Xu J, Li W, Guan Y, et al. MYC/BCL2/BCL6 triple hit and TP53 deletion in a case of high-grade B cell lymphoma receiving CAR T cell immunotherapy. Journal for immunotherapy of cancer. 2021;9(6):e002029.	
Wu Q, Wang F, Yang J, et al. [Identification of TCF3-ZNF384 fusion by transcriptome sequencing in B cell acute lymphoblastic leukemia and its laboratory and clinical characteristics]. Chinese journal of medical genetics. 2021;38(4):351-354. doi:10.3760/cma.j.cn511374-20200416-00273.	
Xu B, Wang D, Huang M, Xiao Y. Sequential therapy of CD19 and CD22 chimeric antigen receptor T cells for relapsed/refractory mediastinal B lymphoblastic lymphoma: Report of one case and review of literature. Journal of Leukemia and Lymphoma. 2021;30(2):91-94.	
Zhang R, Xiao J, Liu Z, Sun Y, Tu S, Li Y, et al. CAR2.0 Therapy for the Management of Post-Transplantation Relapse of B-Cell Acute Lymphoblastic Leukemia. Blood. 2020;136(Supplement 1):7–7.	
Zheng W, Xue Q, Sha X, Wang Y, Wang Y, Liu J, et al. Successful PD-1 inhibitor treatment in a patient with refractory transformed follicular lymphoma who failed to respond to CAR-T cell therapy: a case report and literature review. Cancer biology & therapy. 2021;22(10-12):537–43.	
An open-label, single-center and single-arm clinical study of sequential infusion of anti-CD22 and anti-CD19 CAR-T cells for patients with relapsed or refractory B-cell malignancies. ChiCTR-OPN-16008526. 2016.	Protocol
An open lable, single center, single arm clinical study of infusion of anti-CD19 CAR-T and anti-CD22 CAR-T therapy after autologous hematopoietic stem cell transplantation (auto-HSCT) for relapsed, refractory and high-risk B cell lymphoma. ChiCTR-OPN-16009847. 2016.	
Barrett DM. Getting the most from your CAR target. Blood. 2018;132(14):1467–8.	Wrong intervention
Chen HR, Zhang Y, Chen P, et al. [Efficacy of CD19 Chimeric Antigen Receptors T Cells in the Treatment of Relapsed Patients with B Cell Acute Lymphoblastic Leukemia after Allogeneic Hematopoietic Stem Cell Transplantation]. Zhongguo Shi Yan Xue Ye Xue Za Zhi. 2019;27(4):1040-1045. doi:10.19746/j.cnki.issn.1009-2137.2019.04.008	
Chen X, Yuan L, Zhou J, Wang F, Zhang Y, Ma X, et al. Sustained remission after ruxolitinib and chimeric antigen receptor T-cell therapy bridged to a second allogeneic hematopoietic stem cell transplantation for relapsed Philadelphia chromosome-like B-cell precursor acute lymphoblastic leukemia with novel NPHP3-JAK2 fusion. Genes chromosomes & cancer. 2022;61(1):55–8.	
Guo Y, Feng K, Tong C, Jia H, Liu Y, Wang Y, et al. Efficiency and side effects of anti-CD38 CAR T cells in an adult patient with relapsed B-ALL after failure of bi-specific CD19/CD22 CAR T cell treatment. Cellular & molecular immunology. 2020;17(4):430–2.	
Ma RZ, He Y, Yang DL, Wei JL, Pang AM, Jiang EL, et al. Allogeneic donor-derived CD19 CAR-T therapy of relapsed B-cell acute lmphoblastic leukemia after allogeneic hematopoietic stem cell transplantation. Zhōnghuá xuèyèxué zázhì. 2021;42(5):383–9.	
Marquez CP, Montiel-Esparza R, Hui C, Schultz LM, Davis KL, Hoppe RT, et al. Use of cardiac radiation therapy as bridging therapy to CAR-T for relapsed pediatric B-cell acute lymphoblastic leukemia. Pediatric blood & cancer. 2021;68(3):e28870–n/a.	
Mullanfiroze K, Ottaviano G, Mishra AK, Gabelli M, Lazareva A, Burridge S, et al. Outcomes of Children and Young Adults with Acute Lymphoblastic Leukaemia Administered Inotuzumab Pre CAR-T Therapy. Blood. 2021;138(Supplement 1):1743–1743.	
Staley E, McIntosh E, Hill B. 49 Aberrant Expression of CD5 in the Setting of B-Cell Acute Lymphoblastic Leukemia: A Case Report and Brief Review of the Literature. American journal of clinical pathology. 2018;149(suppl_1):S21–S22.	
Annesley C, Summers C, Pulsipher MA, Wayne AS, Rivers J, Lamble AJ, et al. Clinical Experience of CAR T Cell Immunotherapy for Relapsed and Refractory Infant ALL Demonstrates Feasibility and Favorable Responses. Blood. 2019;134(Supplement_1):3869–3869.	
Borgman P, Ligon J, Yates B, Shalabi H, Foley T, Little L, et al. Abstract PO-111: The impact of race, ethnicity and obesity on CAR T-cell outcomes. Cancer epidemiology, biomarkers & prevention. 2022;31(1_Supplement):PO–111–PO–111.	
Cao XY, Qiu LY, Zhang JP, Xiong M, Zhao YL, Lu Y, et al. CART therapy followed by allo-HSCT for patients with B-cell acute lymphoblastic leukemia relapsing after the first hematopoietic stem cell transplantation. Zhōnghuá xuèyèxué zázhì. 2021;42(4):318–23.	
Deng B, Hu B, Zhang W, et al. Efficacy and safety of sequential administration of car T cells targeting different B cell antigen in children with refractory/relapsed burkitt lymphoma. Abstract Book: 25th Congress of the European Hematology Association Virtual Edition, 2020. HemaSphere. 2020;4(Supplement 1):81.	
Elavia N, McManus A, Highfill SL, Ren J, Shah NN, Fry TJ, et al. The Post-Thaw Recovery of Cryopreserved Chimeric Antigen Receptor (CAR) T-Cells during Manufacture Is Better Than That of Cryopreserved Peripheral Blood CD3+ Cells. Blood. 2017;130:4475–4475.	

Gebre K, Drumheller B, Faude S, Wei J, McFerran J, Pillai V. Absolute Lymphocyte Count Expansion Kinetics Can be Utilized to Assess Response to CAR T Cell Products. Blood. 2021;138(Supplement 1):3874–3874.	Wrong outcomes: report outcomes of multiple CAR T-cell therapies, but do not separate data by CAR T-cell target (CD22 CAR T-cell outcomes unable to be extracted)
Guan Y, Zhang M, Zhang W, Wang J, Shen K, Zhang K, et al. Clinical Utility of Droplet Digital PCR to Monitor BCR-ABL1 Transcripts of Patients With Philadelphia Chromosome-Positive Acute Lymphoblastic Leukemia Post-chimeric Antigen Receptor19/22 T-Cell Cocktail Therapy. Frontiers in oncology. 2021;11:646499–646499.	
Guo J, Zhang Y, Du J. Serum soluble CD25 may predict the early therapeutic response in pediatric patients with B-cell non-Hodgkin's lymphoma(BNHL) during autologous chimeric antigen receptor T cell(CAR-T) therapy. 34th Annual Meeting & Pre-Conference Programs of the Society for Immunotherapy of Cancer (SITC 2019): part 1. Journal for immunotherapy of cancer. 2019;7(S1).	
Holland EM, Yates B, LaLoggia M et al. Characterization of extramedullary disease in all and response to car T-cell therapy. Abstracts - Pediatric blood & cancer. 2021;68(S3).	
Hu K, Yang F, Liu R, Xu T, Zheng P, Feng S, et al. Auto Hematopoietic Stem Cell Transplantation Combined with Another Target Humanized CAR-T Cells for Refractory/Relapsed B-Cell Non-Hodgkin Lymphoma after Failure of Murinized CD19-CAR-T Therapy. Blood. 2021;138(Supplement 1):657.	
Huang H, Jiang Y, Jin Z, Li C, Wu D. Possible Factors on Efficacy and Safety of CAR-T Therapy in Relapsed or Refractory Aggressive B-Cell Lymphoma. Blood. 2018;132(Supplement 1):5386–5386.	
Johnsrud A, Craig J, Baird JH, Spiegel JY, Muffly LS, Zehnder J, et al. Bleeding and Thrombosis Are Associated with Endothelial Dysfunction in CAR-T Cell Therapy and Are Increased in Patients Experiencing Neurologic Toxicity. Blood. 2020;136(Supplement 1):32–3.	
Johnsrud A, Craig J, Baird J, Spiegel J, Muffly L, Zehnder J, et al. Incidence and risk factors associated with bleeding and thrombosis following chimeric antigen receptor T-cell therapy. Blood advances. 2021;5(21):4465–75.	
Li Z, Zheng Q, Wu F, Zhang C, Xu T, Zhao Y, et al. Long-Term Follow-up of Allogeneic Hematopoietic Stem Cell Transplantation after Chimeric Antigen Receptor T-Cell Therapy in Relapsed/Refractory B-Cell Acute Lymphoblastic Leukemia. Blood. 2021;138(Supplement 1):1820–1820.	
Liu S, Deng B, PAN J, Yin Z, Lin Y, Ling Z, et al. Corticosteroids Do Not Influence the Efficacy and Kinetics of CAR-T Cells for B-Cell Acute Lymphoblastic Leukemia. Blood. 2019;134(Supplement_1):228–228.	
Pan J, Xiuwen X, Huilin G, Ling Z, Weiliang S, Xu J, et al. Association of Genetic Factors with Long-Term Outcome in Refractory r/r B-ALL Patients after CAR T-Cell Therapy. Blood. 2019;134(Supplement_1):750–750.	
Parvathaneni K, Toress-Rodriguez K, Meng W, Knox J, Xu X, Weiskopf D, et al. Adoptive Immune Responses to Sars-Cov2 Vaccination in CART19 Treated Patients. Blood. 2021;138(Supplement 1):1757–1757.	
Qu C, Ping N, Wu Q, Kang L, Xia F, Yu L, et al. Radiotherapy Priming Chimeric Antigen Receptor T Cell Therapy Is a Safe and Promising Approach in Relapsed/Refractory Diffuse Large B Cell Lymphoma Patients with High Tumor Burden. Blood. 2018;132(Supplement 1):2961–2961.	
Qu C, Yin J, Kang L, Li Z, Dai H, Zhu X, et al. Successful Treatment of Chimeric Antigen Receptor T Cell Therapy in Refractory or Relapsed Acute Leukemia Patients with TP53 alterations. Blood. 2019;134(Supplement_1):3822–3822.	
Shelikhova L, Molostova O, Rakhteenko A, Khismatullina R, Abugova J, Kurnikova E, et al. Haploidentical Donor-Derived CAR-T Cells Are Safely and Effectively Co-Infused with the Haploidentical Graft, Depleted of Ab T Cells: Report of Case Series. Blood. 2021;138(S1):1736–1736.	
Shi H, Hu K, Ke X, Wu T. Secondary Humanized CAR T Cells Salvage Therapy Improves Survival of R/R DLBCL Patients Failed to Murine CD19-CAR T Cells Therapy. Blood. 2021;138(Supplement 1):4836–4836.	
Silbert SK, Holland EM, Steinberg SM, Little L, Foley T, Shalabi H, et al. Adverse Events Following CAR T-Cell Therapy: A Single Institution Retrospective Analysis of Toxicities Following CAR T-Cell Therapy for Children and Young Adults with Relapsed Refractory B-Cell Acute Lymphoblastic Leukemia. Blood. 2021;138(Supplement 1):1731–1731.	
Song Y, Li Z, Zhao Y, Yang F, Yuan E, Wang Q, et al. Allogeneic-Hematopoietic Stem Cell Transplantation Is Effective and Safe for Relapsed/ Refractory B-ALL Patients Who Cannot Get Remission or MRD Negative after CART Treatment. Blood. 2021;138(Supplement 1):1843–1843.	
Song Y, Pan J, Li Z, Wu T. PD-1 Inhibitor significantly increases acute graft-versus-host disease after allogeneic hematopoietic stem cell transplantation in pediatric refractory/relapsed b-cell acute lymphoblastic leukemia. EHA2021 Virtual Congress Abstract Book. HemaSphere. 2021;5(S2):624-625.	
Tu S, Huang R, Deng L, Zhou X, He Y, Zhou W, et al. Therapy of 4s Chimeric Antigen Receptor T Cells Achieves Long-Term Disease-Free Survival with No Severe CRS or Cres in Patients with Relapsed and Refractory Acute Lymphoblastic Leukemia. Blood. 2018;132(Supplement 1):2696–2696.	
Wang J, Yuan J jun, Mu J, Li Q, Mou N, Yang Z xing, et al. Humanized CD19 CAR-T Cell Combined with Multiple Combination Treatments in Relapsed/Refractory B-Cell Non-Hodgkin Lymphoma. Blood. 2019;134(Supplement_1):5346–5346.	
Xue F, Hu K. Combined treatment of autologous hematopoietic stem cells transplantation with car T-cell therapy improves outcomes in management of advanced relapsed/refractory central nervous system B-cell lymphoma. EHA2021 Virtual Congress Abstract Book. HemaSphere. 2021;5(S2):231-232.	
Zhang J, Yang J, Zhang X, Li J, Cao X, Zhao Y, et al. Significant Long-Term Benefits of CAR T-Cell Therapy Followed By a Second Allo-HSCT for Relapsed/Refractory (R/R) B-Cell Acute Lymphoblastic Leukemia (B-ALL) Patients Who Relapsed after an Initial Transplant. Blood.	

2020;136(Supplement 1):40–1.	
Zhang R, Li Y, Tu S, Huang R, Lai X, Zhang L, et al. Improved Safety and Efficacy of a Multi-Target Chimeric Antigen Receptor Modified T Cell Therapy (4SCAR2.0) Against Relapsed or Refractory Lymphomas. Blood. 2020;136(Supplement 1):47–47.	
Zhang W, Hu B, Jing L, Yang J, Wang S, Liu Y, et al. Early Response Observed in Pediatric Patients with Refractory/Relapsed B-Cell Non-Hodgkin Lymphoma Treated with Sequential Chimeric Antigen Receptor T Cells. Blood. 2019;134(Supplement_1):1945–1945.	
Zhang WQ, Hu B, Jing L, Yang J, Du J, Wang S, et al. PF299 EARLY RESPONSE OBSERVATION OF PEDIATRIC PATIENTS WITH BURKITT'S LYMPHOMA TREATED WITH CHIMERIC ANTIGEN RECEPTOR (CAR) T-CELLS. HemaSphere. 2019;3(S1):101–2.	
Zheng P, Shi H, Hu K, Ke X. Bendamustine is a favorable lymphodepleting chemotherapy regimen prior to car t cells immunotherapy as fludarabine. EHA2021 Virtual Congress Abstract Book. HemaSphere. 2021;5(SUPPL 2):686.	
Zhu J, Yin X, Qin S. Radiotherapy in Combination with Chimeric Antigen Receptor T Cell Therapy Is a Safe and Promising Approach in Relapsed/Refractory Diffuse Large B Cell Lymphoma Patients with High Tumor Burden. International journal of radiation oncology, biology, physics. 2019;105(1):S67–S67.	
Srivastava SK, Panch SR, Jin J, Shalabi H, Shah NN, Highfill SL, et al. Abbreviated T-Cell Activation on the Automated Clinimacs Prodigy Device Enhances Bispecific CD19/22 Chimeric Antigen Receptor T-Cell Viability and Fold Expansion, Reducing Total Culture Duration. Blood. 2018;132(Supplement 1):4551–4551.	Wrong outcomes (manufacturing outcomes only)
Li J, Ho J, Ma F, Li Y, Huang C, Liu L. PS1238 AUTOLOGOUS T CELLS MODIFIED BY CD30 & CD22 DUAL SPECIFIC CAR COMBINED SECRETION OF ANTI-PDL1 SCFV IN TREATING PATIENTS WITH RELAPSED AND REFRACTORY HODGKIN LYMPHOMA. HemaSphere. 2019;3(S1):564–.	Wrong patient population
Miao YR, Liu W, Zhong Z, You Y, Tang Y, Li W, et al. Case Report: Multi-Omics Analysis and CAR-T Treatment of a Chronic Myeloid Leukemia Blast Crisis Case 5 Years After the Discontinuation of TKI. Frontiers in oncology. 2021;11.	
Kokalaki EK, Ma B, Ferrari M, Grothier T, Hazelton W, Manzoor S, et al. A High Sensitivity aCD22 CAR Combined with aCD19 CAR to Generate Dual Targeting CAR T Cells for the Treatment of r/r B-ALL. Blood. 2021;138(Supplement 1):1710–1710.	Wrong study design (not a CAR T-cell clinical trial)
Meng Y, Deng B, Rong L, Li C, Song W, Ling Z, et al. Short-Interval Sequential CAR-T Cell Infusion May Enhance Prior CAR-T Cell Expansion to Augment Anti-Lymphoma Response in B-NHL. Frontiers in oncology. 2021;11:640166–640166.	
Wang L, Zhang Y, Anderson E, Kumar R, Lamble AJ, Orentas RJ. CD22 CAR-T Induces Both CD19 and CD22 Surface Down-Modulation: Defining a Mechanism of Generalized Immune Evasion and the Effects of Epigenetic Modifiers. Blood. 2020;136(Supplement 1):22–3.	
Wang L, Hong R, Zhou L, Ni F, Zhang M, Zhao H, et al. New-Onset Severe Cytopenia After CAR-T Cell Therapy: Analysis of 76 Patients With Relapsed or Refractory Acute Lymphoblastic Leukemia. Frontiers in oncology. 2021;11:702644–702644.	
Pan J, Deng B, Ling Z, Song W, Xu J, Duan J, et al. Ruxolitinib mitigates steroid-refractory CRS during CAR T therapy. Journal of cellular and molecular medicine. 2021;25(2):1089–99.	
Zhou Y, Li J, Zhang X, Jia T, Zhang B, Dai N, et al. Prognostic Value of Radiomic Features of 18F-FDG PET/CT in Patients With B-Cell Lymphoma Treated With CD19/CD22 Dual-Targeted Chimeric Antigen Receptor T Cells. Frontiers in oncology. 2022;12:834288–834288.	Included patients with history of CAR T-cells, but cannot include due to risk of double counting patients from other studies
Dai H, Wu Z, Jia H, Tong C, Guo Y, Ti D, et al. Correction to: Bispecific CAR-T cells targeting both CD19 and CD22 for therapy of adults with relapsed or refractory B cell acute lymphoblastic leukemia. Journal of hematology and oncology. 2020;13(1):53–53.	Duplicates
Du J, Zhang Y. Correction to: Sequential anti-CD19, 22, and 20 autologous chimeric antigen receptor T-cell (CAR-T) treatments of a child with relapsed refractory Burkitt lymphoma: a case report and literature review. Journal of cancer research and clinical oncology. 2020;146(8):2177–2177.	
Hua J, Qian W, Wu X, Zhou L, Yu L, Chen S, et al. Sequential Infusion of Anti-CD22 and Anti-CD19 Chimeric Antigen Receptor T Cells for a Pediatric Ph-Like B-ALL Patient That Relapsed After CART-Cell and Haplo-HSCT Therapy: A Case Report and Review of Literature [Corrigendum]. OncoTargets and therapy. 2020;13:3045–6.	
Meng Y, Deng B, Rong L, Li C, Song W, Ling Z, et al. Corrigendum: Short-Interval Sequential CAR-T Cell Infusion May Enhance Prior CAR-T Cell Expansion to Augment Anti-Lymphoma Response in B-NHL. Frontiers in oncology. 2021;11:778039–778039.	
Schultz LM, Davis KL, Baggott C, Chaudry C, Marcy AC, Mavroukakis S, et al. Phase 1 Study of CD19/CD22 Bispecific Chimeric Antigen Receptor (CAR) Therapy in Children and Young Adults with B Cell Acute Lymphoblastic Leukemia (ALL). Blood. 2018;132(Supplement 1):898–898.	
Shah NN, Shalabi H, Yates B, Yuan C, Qin H, Ombrello A, et al. Abstract LB-146: Phase I CD22 CAR T-cell trial updates. Cancer research (Chicago, Ill). 2019;79(13_Supplement):LB–146–LB–146.	
Zhang Y, Chen H, Song Y, Tan X, Zhao Y, Liu X, et al. Chimeric antigens receptor T cell therapy as a bridge to haematopoietic stem cell transplantation for refractory/ relapsed B-cell acute lymphomalastic leukemia. British journal of haematology. 2020;189(1):146–52.	

Targeting CD19–CD22 Aids Younger Patients with ALL. Cancer discovery. 2020;10(8):OF1–OF1.	Commentary, news, editorials, or reviews
Dual CD19/CD22 CAR T Cells Show Feasibility in Pediatric/Young Adult B-ALL. Cancer discovery. 2021;11(12):2958–2958.	
CD19/CD22 Dual-Targeted CAR-T Therapy Active in Relapsed/Refractory DLBCL. The oncologist (Dayton, Ohio). 2020;25(S1):S12–S13.	
CAR T Cells Targeting Two Antigens Show Efficacy in B-cell Malignancies. Cancer discovery. 2021;11(10):2366–2366.	
CAR Linker Length Modulates CD22-Targeted CAR T-cell Efficacy in ALL. Cancer discovery. 2021;11(7):1611–1611.	
Cailly L, Leleu X, Moya N. One CAR can hide another: anti-CD22 CAR-T cells in relapsed or refractory acute b-lymphoblastic leukemia. Hematologie. 2020;26(1):9-10.	
Doan A, Pulsipher MA. Hypogammaglobulinemia due to CAR T-cell therapy. Pediatric blood & cancer. 2018;65(4):e26914–n/a.	
Killock, D. Anti-CD22 CAR T cells in ALL. Nat Rev Clin Oncol 17, 391 (2020). https://doi.org/10.1038/s41571-020-0379-x	
Anti-CD22 CAR Therapy Leads to ALL Remissions. Cancer discovery. 2017;7(2):120–120.	
Shah NN. The one-two punch (of CAR T cells). Blood. 2020;135(5):303–4.	
Wäsch R, Munder M, Marks R. Teaming up for CAR-T cell therapy. Haematologica (Roma). 2019;104(12):2335–6.	
